# QeITH: Quantifies Tumor Ecosystem Heterogeneity to Predict Cancer Progression and Treatment Benefit

**DOI:** 10.34133/csbj.0061

**Published:** 2026-06-18

**Authors:** Qiqi Lu, Jiangti Luo, Jiawei Wang, Bangqi Zhao, Xiaosheng Wang, Linjun You

**Affiliations:** ^1^ Biomedical Informatics Research Lab, School of Basic Medicine and Clinical Pharmacy, China Pharmaceutical University, Nanjing 211198, China.; ^2^ Department of Clinical Pharmacy, School of Basic Medicine and Clinical Pharmacy, China Pharmaceutical University, Nanjing 211198, China.; ^3^ Center for Precision Health, McWilliams School of Biomedical Informatics, The University of Texas Health Science Center at Houston, Houston, TX 77030, USA.

## Abstract

Intratumor heterogeneity (ITH) is a fundamental driver of therapeutic failure and disease progression. However, the complexity of the tumor ecosystem is a critical yet underexplored aspect, making its precise quantification essential for fully deciphering ITH and its clinical implications. To address this, we developed Quantifying Ecosystem Intratumor Heterogeneity (QeITH), a computational framework that applies Shannon entropy to quantify ecosystem heterogeneity by measuring the diversity and distributional entropy of cellular compositions and functional states across single-cell, bulk, and spatial transcriptomics. At the single-cell resolution, QeITH identifies elevated ITH as intrinsic markers of malignant transformation, yet enhanced sensitivity to therapy. Pan-cancer bulk analyses further link elevated QeITH scores to increased neoantigen burden, *PD-L1* expression, and unfavorable prognosis. Notably, spatial transcriptomics reveals that ecological complexity is nonuniformly distributed, peaking at invasive fronts and within tertiary lymphoid structures (TLS), where enhanced diversity within TLS modulates therapeutic vulnerability. Thus, QeITH reveals a dual role for ITH: While high scores associated with tumor aggressiveness, they also predict favorable treatment responses by capturing an immunologically active tumor ecosystem state. By integrating single-cell precision with spatial context, this framework elucidates the biological drivers of cancer progression and serves as a robust tool for optimizing personalized therapeutic strategies in precision oncology.

## Highlights

• QeITH framework for ecosystem heterogeneity quantification: The QeITH framework quantifies ecosystem-level intratumor heterogeneity by measuring the diversity and distributional entropy of cellular compositions and functional states within the tumor microenvironment.
• Dual clinical implications of high QeITH: Elevated QeITH scores are associated with poor long-term prognosis but also predict favorable responses to therapies, indicating an immunologically active tumor ecosystem.
• Ecological complexity and spatial dynamics: QeITH reveals that high ecological complexity correlates with tumor aggressiveness features and is unevenly distributed within the tumor. This provides valuable insights into the spatial dynamics of ITH.
• Open source: QeITH is implemented with a publicly available R package, promoting accessibility for further research.

## Graphical Abstract

**Figure:**
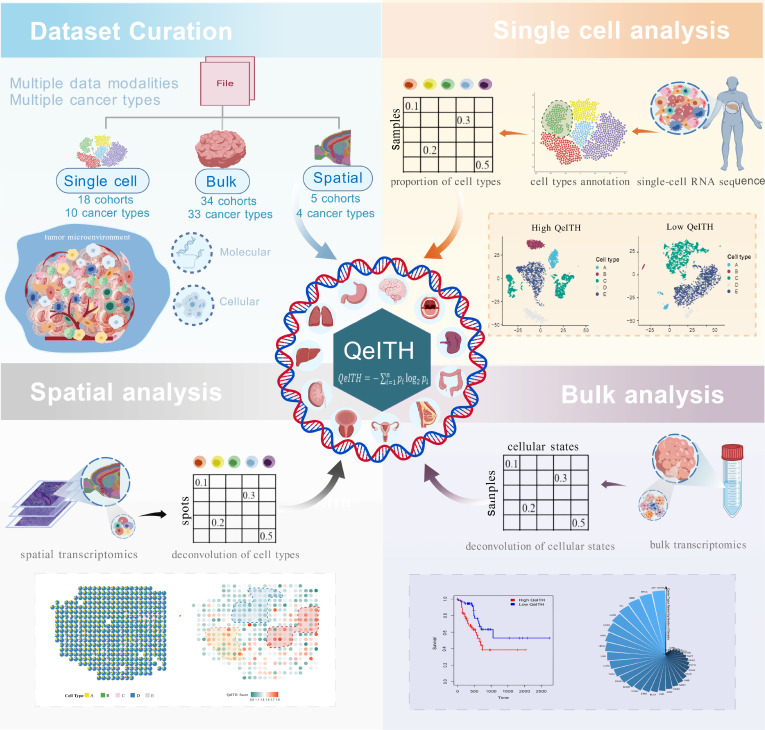

## Introduction

Intratumor heterogeneity (ITH) is a fundamental hallmark of cancer, characterized by the complex coexistence of genetically diverse subclones and distinct cell states within a tumor [1]. As a primary driver of tumor evolution, therapeutic resistance, and disease progression, the accurate quantification of ITH is indispensable for advancing precision oncology [2,3]. Traditionally, ITH has been evaluated across multiple omics profiles, including genomics [4–7], transcriptomics [8], proteomics [9], and epigenomics [10]. For instance, entropy-based frameworks such as DITHER have been developed to quantify genomic heterogeneity [7], while other approaches have applied entropy measures to bulk transcriptomics [11] and spatial imaging data [12] to capture distinct facets of tumor complexity [13]. At the single-cell resolution, ROGUE have been developed to quantify the purity of identified cell clusters, where 1-ROGUE represents transcriptional heterogeneity within clusters [14]. However, existing computational frameworks primarily focus on the heterogeneity of malignant cells, largely overlooking the intricate heterogeneity inherent in the broader tumor microenvironment (TME).

While traditional bulk sequencing provides a global molecular level, it fails to capture the high-resolution cellular diversity and spatial architecture of the TME [15]. The TME is a complex ecosystem comprising cancer cells, immune cells, and stromal cells, alongside vascular components, all of which collectively modulate tumor behavior and clinical outcomes [16,17]. Although recent breakthroughs in single-cell RNA sequencing (scRNA-seq) and spatial transcriptomics have provided unprecedented resolution into these cellular landscapes [18,19], computational tools capable of assessing the heterogeneity of the total tumor ecosystem across multiple data modalities remain scarce. Moreover, existing methods are largely confined to specific data types or cellular compartments, limiting our ability to integrate insights across scales—from single-cell resolution to tissue architecture—and to capture the ecological complexity that emerges from interactions between malignant, immune, and stromal components.

Recent efforts have begun to quantify specific aspects of ITH. Our group recently introduced ScImTH (scoring immunological intratumor heterogeneity) to quantify immune cell diversity within the TME and demonstrated its important prognostic and predictive value [20]. While ScImTH focused specifically on the immune compartment, we recognized the need for a more comprehensive measure of tumor ecosystem complexity. To address this gap, we developed QeITH (quantifying ecosystem intratumor heterogeneity), a universally applicable computational framework designed to quantify the ecosystem heterogeneity index across single-cell, bulk, and spatial resolutions. In contrast to established genomic ITH metrics, which quantify genetic subclonal diversity arising from somatic mutations and copy number alterations within malignant cells, QeITH is designed as an ecosystem heterogeneity index. Rather than focusing solely on cancer cell-intrinsic heterogeneity, QeITH captures the diversity and distribution of all cellular components within the TME, including malignant cells, immune cells, and stromal cells, as well as their functional states. This conceptual distinction is fundamental: While genomic ITH metrics reflect the evolutionary dynamics of cancer clones, QeITH quantifies the ecological complexity of the tumor as a whole—a dimension of heterogeneity that is largely orthogonal to genetic diversity but critically shapes tumor behavior and therapeutic response. We validated QeITH across an extensive spectrum of cancer types and data modalities, systematically evaluating its associations with key molecular features and clinicopathological outcomes. This metric provides novel perspective on the complex interplay between tumor ecosystem diversity and cancer biology, offering a robust tool for deciphering the drivers of malignancy.

## Methods

### Overview of the QeITH algorithm

We developed the QeITH algorithm to quantify ITH by calculating the Shannon entropy of the tumor’s ecological composition. The score captures both the diversity and evenness of the cellular landscape. The ITH score is defined as:

$$\text{QeITH}=-\sum_{i=1}^{n}p_{i}{\text{log}}_{2}p_{i},$$ (1)

where *n* is the number of distinct cell types or states, and $p_{i}$ denotes the relative proportion of the *i*th component, with $\sum_{i=1}^{n}\;p_{i}=1$. We applied this algorithm across 3 data modalities:

1. scRNA-seq data: $p_{i}$ is defined as the proportion of a specific cell type relative to the total number of cells in the sample.
2. Spatial transcriptomics: $p_{i}$ is derived from the deconvoluted proportions of cell types within each spatial spot or segment.
3. Bulk transcriptomics: $p_{i}$ represents the relative proportion of 14 tumor or TME-associated functional states [angiogenesis, apoptosis, cell cycle, differentiation, DNA damage, DNA repair, epithelial–mesenchymal transition (EMT), hypoxia, inflammation, invasion, metastasis, proliferation, quiescence, and stemness] [21].

**Fig. 1.**
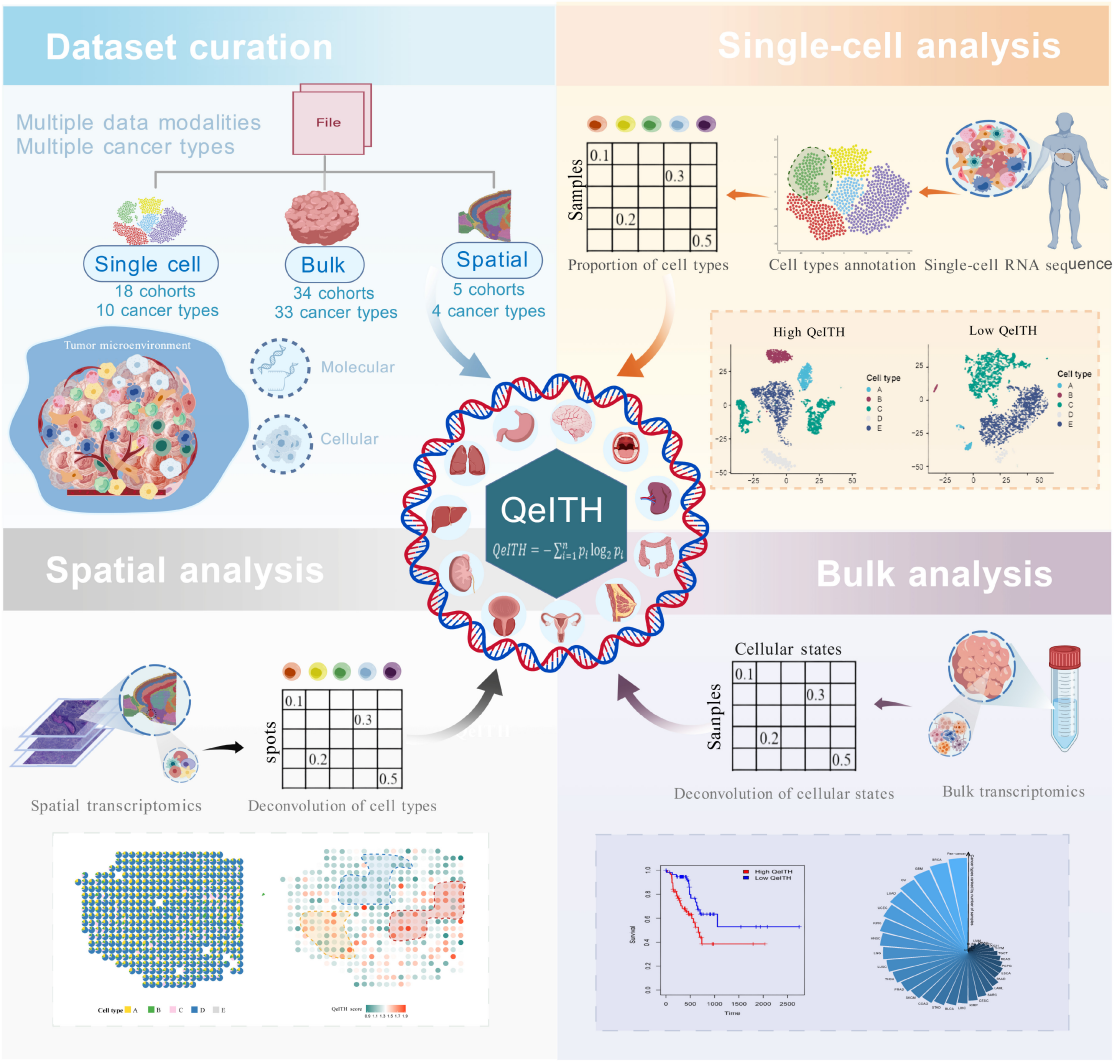
Illustration of the QeITH algorithm to quantify ITH. The figure was created with BioGDP.com [57].

An overview of the workflow is shown in Fig. 1. The algorithm is implemented as an R package, publicly available at https://github.com/XS-Wang-Lab/QeITH. The web application of QeITH is available at the website: https://xs-wang-lab.shinyapps.io/qeith-shiny.

### Deconvolution of spatial transcriptomics data

Spatial transcriptomics datasets were deconvolved using the CARD algorithm [22] to estimate cell type proportions at each spot, utilizing matched scRNA-seq dataset as the references. Analyses were implemented using the CARD R package. For each dataset, a CARD object was created using the createCARDObject function with the following parameters: the spatial count matrix and spatial coordinates, the single-cell count matrix and cell type metadata as reference, and quality control thresholds of minCountGene = 100 and minCountSpot = 5 to filter out genes and spots with insufficient counts. Deconvolution was then performed using the CARD_deconvolution function with default parameters.

### Quantification of functional state proportions from bulk transcriptomics

To quantify the functional heterogeneity of the tumor ecosystem, we selected 14 functional states closely related to tumor biology and the TME. The gene signatures for each functional state were obtained from previously published study, and the complete gene lists are provided in Table S1. For bulk transcriptomics data, the relative proportion $p_{i}$ for each of the 14 tumor or TME-associated functional states was derived using a systematic approach. Single-sample gene set enrichment analysis (ssGSEA) scores for each functional state were calculated per sample using the GSVA R package [23], along with its curated gene signatures. To transform these scores into a format suitable for entropy calculation, we first truncated negative values to zero, based on the rationale that a negative enrichment score reflects a functionally inactive state in that sample. The proportion $p_{i}$ for the *i*th state within a sample was then defined as the non-negative ssGSEA score divided by the sum of all 14 non-negative ssGSEA scores for that sample, ensuring $\sum_{i=1}^{n}\;p_{i}=1$. This approach quantifies functional diversity using relative activation levels of different states.

To assess the robustness of QeITH to methodological choices, we conducted 3 sensitivity analyses. First, we compared the original zero-thresholding approach for handling negative ssGSEA values to a global shift method (adding the minimum value across all samples). Second, we evaluated alternative normalization schemes: rank-based quantile normalization and square normalization, alongside the original probability normalization. Third, we tested the stability of QeITH to gene set composition by randomly subsampling 6 to 13 gene sets from the original 14 (100 iterations per subset size) and recalculating ITH scores. For each alternative method, we computed Spearman correlations between the derived ITH scores and the original ITH scores across the pan-cancer cohort.

### Datasets

We downloaded 12 scRNA-seq datasets from National Center for Biotechnology Information (NCBI) Gene Expression Omnibus (GEO) (https://www.ncbi.nlm.nih.gov/geo/). These datasets include GSE176078 (breast cancer) [24], GSE263733 (pancreatic cancer) [25], GSE118828 (ovarian cancer) [26], GSE131907 (lung cancer) [27], GSE181919 (head and neck cancer) [28], GSE149614 (hepatocellular carcinoma) [29], GSE266577 (ovarian cancer) [30], GSE166555 (colorectal cancer) [31], GSE278688 (pancreatic cancer) [32], GSE232316 (glioma cancer) [33], GSE206785 (gastric cancer) [34], and GSE210347 (pan-cancer) [35]. In addition, we sourced 1 lung cancer scRNA-seq dataset [36], 2 breast cancer scRNA-seq datasets [37,38], 1 pancreatic cancer scRNA-seq dataset [39], 1 hepatocellular carcinoma scRNA-seq dataset [40], and 1 kidney cancer scRNA-seq dataset [41] from their respective publications. All datasets were acquired as gene expression matrices paired with corresponding cell annotation metadata. Furthermore, we obtained gene expression profiles [RNA-seq by expectation-maximization (RSEM) normalized] and clinical data for 33 cancer types from The Cancer Genome Atlas (TCGA) through the UCSC Xena platform (https://xenabrowser.net/datapages/). These cancer types included adrenocortical carcinoma (ACC), bladder urothelial carcinoma (BLCA), breast invasive carcinoma (BRCA), cervical squamous cell carcinoma and endocervical adenocarcinoma (CESC), cholangiocarcinoma (CHOL), colon adenocarcinoma (COAD), lymphoid neoplasm diffuse large B cell lymphoma (DLBC), esophageal carcinoma (ESCA), glioblastoma multiforme (GBM), head and neck squamous cell carcinoma (HNSC), kidney chromophobe (KICH), kidney renal clear cell carcinoma (KIRC), kidney renal papillary cell carcinoma (KIRP), acute myeloid leukemia (LAML), brain lower grade glioma (LGG), liver hepatocellular carcinoma (LIHC), lung adenocarcinoma (LUAD), lung squamous cell carcinoma (LUSC), mesothelioma (MESO), ovarian serous cystadenocarcinoma (OV), pancreatic adenocarcinoma (PAAD), pheochromocytoma and paraganglioma (PCPG), prostate adenocarcinoma (PRAD), rectum adenocarcinoma (READ), sarcoma (SARC), skin cutaneous melanoma (SKCM), stomach adenocarcinoma (STAD), testicular germ cell tumors (TGCT), thyroid carcinoma (THCA), thymoma (THYM), uterine corpus endometrial carcinoma (UCEC), uterine carcinosarcoma (UCS), and uveal melanoma (UVM). Additionally, we downloaded a large-scale breast cancer transcriptomic dataset (Metabric-BRCA [42]) from cBioPortal (www.cbioportal.org/). We acquired transcriptomic datasets and corresponding immunotherapy response information from 3 independent SKCM cohorts [43–45] and one non-small cell lung cancer (NSCLC) [46]. Finally, we obtained 2 processed spatial transcriptomic datasets for breast cancer [24,47] and a matched scRNA-seq reference dataset [24]. Additionally, we acquired spatial transcriptomic datasets for glioblastoma [48], pancreatic cancer [49], and renal cell carcinoma [50]. A full summary of all datasets is provided in Table S2.

### scRNA-seq data analysis

We analyzed scRNA-seq data using the Seurat R package [51]. For each dataset, raw count matrices were loaded using the CreateSeuratObject function with min.cells = 0 and min.features = 0 to preserve the original data structure as provided by the authors. Data were then log-normalized using the NormalizeData function with a scale factor of 10,000, and the top 2,000 highly variable genes were identified using the FindVariableFeatures function with the “vst” selection method. Prior to dimensionality reduction, data were scaled using the ScaleData function based on the top 2,000 highly variable genes. Principal components analysis (PCA) was performed using the RunPCA function based on the identified highly variable genes, with 50 principal components computed. For nonlinear dimensionality reduction and visualization, we applied uniform manifold approximation and projection (UMAP) using the RunUMAP function based on the first 15 principal components. The resulting low-dimensional embeddings were visualized using the DimPlot function. We directly used the cell type annotation files provided by the original authors. For datasets where only sample-level cell type proportions were available rather than single-cell annotations, we directly used the provided proportion matrices as input for QeITH. The annotation files were downloaded from public repositories and integrated into the Seurat object using the AddMetaData function. This approach ensures consistency with the original studies. For datasets generated from multiple batches or samples, we confirmed that batch correction had been performed in the original studies. Therefore, no additional batch correction was applied in our analysis.

### Survival analysis

Survival differences among patient groups were evaluated using the Kaplan–Meier (KM) method [52] and visualized using KM curves. The log-rank test was employed to assess statistical significance between patients with high ITH score (top third) and those with low ITH score (bottom third). To evaluate the independent prognostic value of the ITH score, multivariable Cox proportional hazards regression was conducted using the coxph function. Continuous variables [tumor mutational burden (TMB), tumor purity, immune score, and ITH score] were standardized prior to analysis. The proportional hazards assumption was tested using Schoenfeld residuals. All survival analyses were implemented using the survival R package.

### Evaluation of ITH score variability

We assessed the variation of ITH scores within each cancer type using the median absolute deviation (MAD). For a given cancer type, the MAD was calculated as:

$$\mathrm{MAD}=\text{median}(|x_{i}-\tilde{x}|)$$ (2)

where $x_{i}$ represents the ITH score of the *i*th tumor and $\tilde{x}$ denotes the median ITH score of the cohort.

### Comparison with other ITH algorithms

To benchmark our method, we compared QeITH with existing algorithms. Mutant-allele tumor heterogeneity (MATH) scores were calculated using the math.score function from the maftools R package [4] using mutation annotation format (MAF) files. ABSOLUTE scores (specifically tumor ploidy) [5] were derived from SNP6 array data. Both MAF and SNP6 files were obtained from the Genomic Data Commons (GDC) Data Portal (https://portal.gdc.cancer.gov/). Additionally, we obtained pre-calculated ITH scores for TCGA cohorts by DEPTH2 [8], PhyloWGS [6], and DITHER [7] directly from their respective publications. For single-cell validation, we compared QeITH with ROGUE, for which 1-ROGUE represents transcriptional heterogeneity within clusters. Sample-level heterogeneity scores were calculated as the mean of (1-ROGUE) across all clusters in each sample.

### Evaluation of TME and neoantigens

Immune scores, stromal scores, and tumor purity were estimated using the ESTIMATE algorithm [13] based on gene expression profiles. Neoantigen load data for TCGA cohorts were obtained from Thorsson et al. [53].

### Statistical analysis

For non-normally distributed data, we used the Mann–Whitney *U* test to compare 2 groups and the Kruskal–Wallis test for more than 2 groups. Correlations between ITH scores and other variables were assessed using Spearman rank correlation, reporting correlation coefficients (*ρ*) and *P* values. Sequential nested models were constructed to assess the incremental predictive value of QeITH beyond established prognostic or response factors. For survival outcomes, Cox proportional hazards models were used; for response outcomes, multivariable logistic regression models were used. All continuous variables were standardized (*z*-score normalization) prior to model fitting to facilitate comparison of effect sizes across covariates. Model fit was compared using likelihood ratio tests (LRTs) and *P* values. To control for multiple testing, *P* values were adjusted using the Benjamini–Hochberg false discovery rate (FDR) method, and only results with FDR < 0.05 were considered significant. To assess the spatial organization of ITH scores, we performed global spatial autocorrelation analysis using Moran’s *I* with *k*-nearest neighbors (k = 6) and permutation tests (9,999 iterations) using the spdep package in R.

### Software and hardware

All analyses were performed using R software (versions 4.2.2 and 3.6.1). Key R packages, their versions used, and the parameter settings for each analysis step in this study are described in the corresponding sections above and summarized in Table S5.

## Results

### Overview of the QeITH algorithm

The QeITH algorithm quantifies ITH for single-cell, bulk, or spatial transcriptomics data using gene expression matrices (Fig. 1). For single-cell transcriptomics, QeITH computes an ITH score for each tumor directly based on the abundance of annotated cell types. For bulk transcriptomics, the algorithm quantifies ITH by calculating the informational entropy of the relative proportions of 14 tumor-associated functional states, reflecting the diversity and distribution of the TME. For spatial transcriptomics, the ITH level is estimated for each specific spot or region within a tumor based on the proportions of identified cell types. We validated QeITH across single-cell, bulk, and spatial datasets in both pan-cancer and individual cancer types. Hereafter, we refer to the metric quantified by QeITH as the “ITH score”.

### Elevated ITH scores are associated with tumor progression phenotypes

**Fig. 2.**
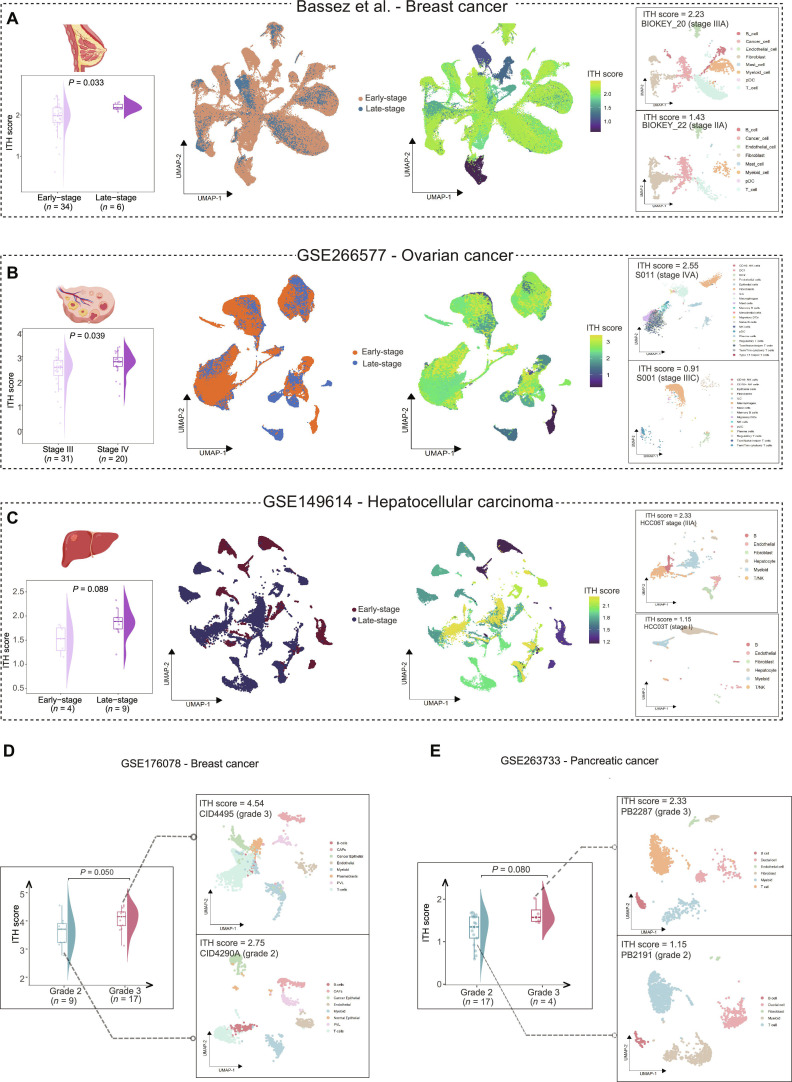
Elevated ITH scores are associated with tumor progression phenotypes in single-cell datasets. (A to C) Raincloud plots showing ITH score distributions in breast cancer (A), ovarian cancer (B), and hepatocellular carcinoma (C). Early-stage tumors exhibited significantly lower ITH scores than late-stage tumors across all 3 cancer types. Boxplots with overlaid jittered points show median, interquartile range (IQR), and individual sample values. Half-violins on the side show density distributions of ITH scores. For each cancer type, sample-level metrics (stage, ITH score) are mapped to all cells from each sample. UMAP visualizations are shown: the left UMAP shows cells colored by tumor stage (early versus late); the middle UMAP shows cells colored by ITH score (blue to red); the right UMAP panels show cell type distributions for a high-ITH sample (top) and a low-ITH sample (bottom). (D and E) Raincloud plots showing ITH score distributions in breast cancer (D) and pancreatic cancer (E) stratified by tumor grade. Grade 3 tumors exhibited significantly higher ITH scores compared to grade 2 tumors. Boxplots with overlaid jittered points show median, IQR, and individual sample values. Half-violins on the side show density distributions of ITH scores. For each cancer type, the UMAP panels show cell type distribution for a high-ITH sample (top) and a low-ITH sample (bottom). The 2-tailed Mann–Whitney *U* test *P* values are shown. *P < 0.05; **P < 0.01; *** *P* < 0.001; ns, not significant. It also applies to the following figures.

Our analysis revealed marked associations between ITH scores and varying clinicopathological features and risk factors. In breast cancer, we observed consistent patterns of ITH progression. The Bassez et al. cohort [38] demonstrated that early-stage tumors (stage IA/IIA/IIB) exhibited significantly lower ITH scores compared to late-stage tumors (stage IIIA/IIIC) (*P* = 0.033; Fig. 2A), as exemplified by BIOKEY_20 versus BIOKEY_22. Similar trends were observed in other malignancies. In ovarian cancer [30], advanced-stage tumors (stage IV) demonstrated elevated ITH scores compared to stage III tumors (*P* = 0.039; Fig. 2B), indicating that metastatic progression may be associated with increased clonal diversity. In hepatocellular carcinoma [29], late-stage tumors (stage IIIA/IIIB/IV) exhibited higher ITH than early-stage tumors (stage I/II) (*P* = 0.089; Fig. 2C). Notably, the relationship between ITH and tumor grade was remarkably consistent; grade 3 tumors exhibited significantly higher ITH scores compared to grade 2 tumors in both breast cancer [24] (*P* = 0.050; Fig. 2D and Fig. S1A) and pancreatic [25] (*P* = 0.080; Fig. 2E and Fig. S1B) cohorts. These recurring patterns underscore that increasing ITH is a common feature of disease progression.

### Associations with clinical features and therapy response

**Fig. 3.**
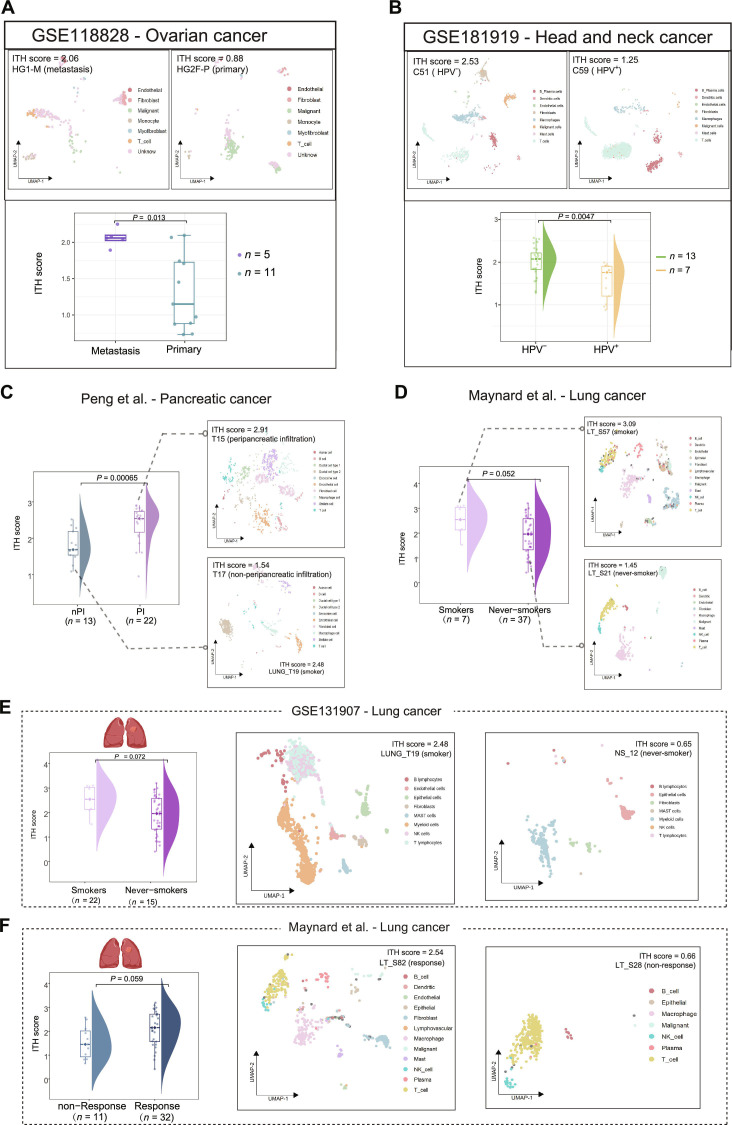
Associations with clinical features and therapy response in single-cell datasets. (A) In ovarian cancer, metastatic lesions harbored significantly higher ITH scores than primary tumors. The top UMAP panels show cell type distribution for a high-ITH sample (left) and a low-ITH sample (right). The bottom boxplots with jittered points show IQR and individual sample values. (B) HPV-negative head and neck cancers showed markedly elevated ITH scores compared to HPV-positive cases. The UMAP visualizations are shown: the left UMAP shows cell distributions for high ITH samples; the right UMAP shows cell distributions for low ITH samples. The bottom panel shows a raincloud plot comparing ITH score distributions between HPV-negative and HPV-positive cases. Boxplots with overlaid jittered points show median, IQR, and individual sample values. Half-violins on the side show density distributions of ITH scores. (C) Pancreatic tumors with peripancreatic infiltration (PI) had higher ITH scores than those without infiltration. (D) Lung cancer patients with smoking history demonstrated higher ITH scores than never-smokers in the lung cancer from the Maynard et al. dataset. For both (C) and (D), boxplots with overlaid jittered points show median, IQR, and individual sample values. Half-violins on the side show density distributions of ITH scores. For each panel, the UMAP visualizations show cell type distributions for a high-ITH sample (top) and a low-ITH sample (bottom). (E) Lung cancer patients with smoking history demonstrated higher ITH scores than never-smokers (Kim et al.). (F) In lung cancer, targeted therapy responders (complete response/partial response) exhibited significantly higher ITH scores than nonresponders (stable disease/progressive disease). For both (E) and (F), boxplots with overlaid jittered points show median, IQR, and individual sample values. Half-violins on the side show density distributions of ITH scores. For each panel, the UMAP visualizations show cell type distributions for a high-ITH sample (middle) and a low-ITH sample (right). The 2-tailed Mann–Whitney *U* test *P* values are shown.

In advanced-stage tumors, the association between ITH and key clinicopathological characteristics became increasingly evident. Analysis of ovarian cancer [26] revealed that metastatic lesions harbored significantly higher ITH scores compared to primary tumors (*P* = 0.013; Fig. 3A and Fig. S2A). In head and neck cancer [28], human papillomavirus (HPV)-negative tumors exhibited markedly elevated ITH relative to HPV-positive cases (*P* = 0.0047; Fig. 3B and Fig. S2B), aligning with the established clinical understanding that HPV-positive tumors generally have a more favorable prognosis [54]. Furthermore, in pancreatic cancer [39], peripancreatic infiltration—a marker of local invasion—was associated with markedly increased ITH (*P* = 6.5 × 10^−4^; Fig. 3C and Fig. S2C), suggesting potential evolutionary dynamics during invasive progression.

Regarding lifestyle and treatment factors, current smokers in a lung cancer cohort [36] demonstrated higher ITH scores compared to never-smokers (*P* = 0.052; Fig. 3D and Fig. S2D), a trend mirrored in another independent LUAD cohort [27] (*P* = 0.072; Fig. 3E and Fig. S2E). Notably, in the lung cancer cohort reported by Maynard et al. [36], patients who responded to targeted therapy exhibited significantly higher ITH scores than nonresponders (*P* = 0.059; Fig. 3F and Fig. S2F). This suggests that the overall cellular composition of high ITH tumors, which includes both diverse subclones and immune cell populations, may contribute to the heterogeneous responses observed with targeted therapy.

### ITH correlates with malignant transformation and subtype aggressiveness

**Fig. 4.**
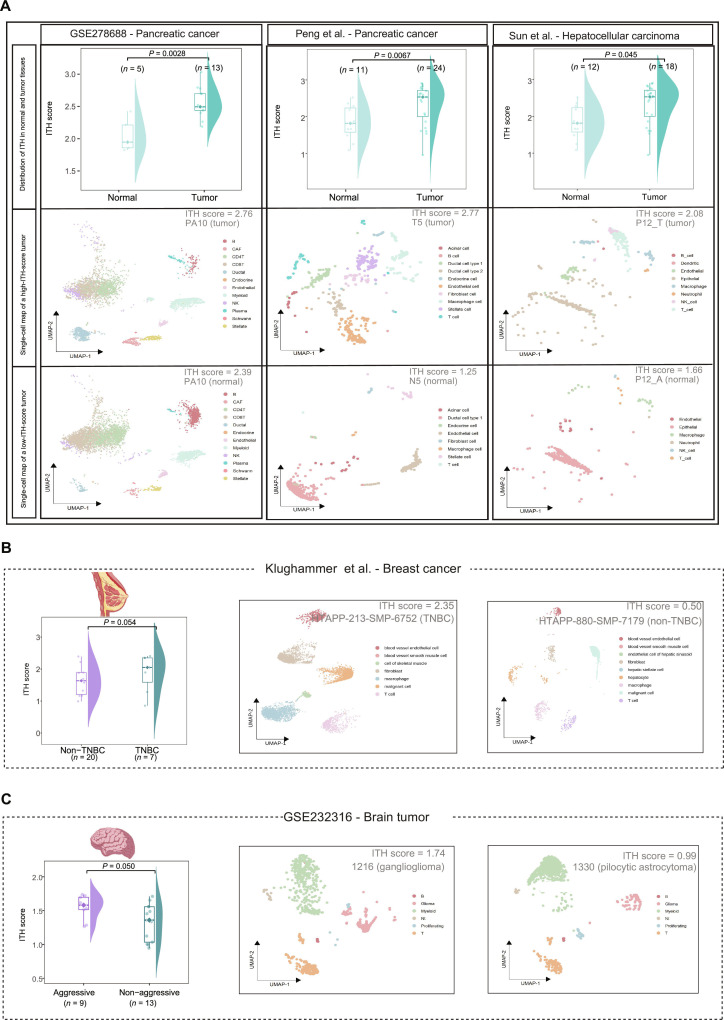
ITH scores correlate with malignant transformation and subtype aggressiveness in single-cell datasets. (A) ITH scores were elevated in tumors versus normal tissues across 2 pancreatic cancer cohorts and a hepatocellular carcinoma dataset. The top boxplots with jittered points show IQR and individual sample values. The UMAP panels show cell type distribution for a high-ITH sample (middle) and a low-ITH sample (bottom). (B) Triple-negative breast cancer (TNBC) exhibited markedly elevated ITH scores compared to non-TNBC. (C) Pilocytic astrocytomas demonstrated notably lower ITH scores than the more progressive gangliogliomas and low-grade gliomas. For both (B) and (C), boxplots with overlaid jittered points show median, IQR, and individual sample values. Half-violins on the side show density distributions of ITH scores. For each panel, the UMAP visualizations show cell type distributions for a high-ITH sample (middle) and a low-ITH sample (right). The 2-tailed Mann–Whitney *U* test *P* values are shown.

Our findings establish the ITH score as a robust and effective bioinformatics metric for distinguishing disease state and facilitating subtype stratification across diverse cancer types. Tumor tissues consistently harbored markedly elevated ITH scores compared to normal counterparts. This distinction was evident in 2 independent pancreatic cancer cohorts [32,39], where tumor specimens exhibited markedly higher ITH scores compared to normal tissues (*P* = 2.8 × 10^−3^ and *P* = 6.7 × 10^−3^, respectively; Fig. 4A). This pattern’s generalizability was confirmed across gastrointestinal malignancies, including hepatocellular carcinoma [40] (*P* = 0.045; Fig. 4A), colorectal cancer [31] (*P* = 0.018), kidney cancer [41] (*P* = 1.4 × 10^−4^), and gastric cancer [34] (*P* = 0.006; Fig. S3A). A subsequent pan-cancer analysis revealed a progressive increase in ITH scores from normal to tumor tissues [35] (*P* = 0.010; Fig. S3A), underscoring its role in tumorigenesis.

Striking differences in the ITH score were also evident when comparing different cancer subtypes. In breast cancer [37], triple-negative breast cancer (TNBC), known for its aggressive behavior, exhibited markedly elevated ITH scores compared to non-TNBC (*P* = 0.054; Fig. 4B). Similarly, in kidney cancer [41], the more aggressive KIRC had significantly higher ITH scores than the more indolent KIRP (*P* = 0.096; Fig. S3B). In brain tumors [33], the clinically indolent pilocytic astrocytomas demonstrated notably lower ITH scores than the more progressive gangliogliomas and low-grade gliomas (*P* = 0.026; Fig. 4C).

These results collectively position ITH not only as a biomarker of malignant transformation but also as a potential indicator of intra-tumor phenotypic diversity, with implications for understanding tumor evolution and subtype-specific therapeutic strategies.

### Robustness and benchmarking of QeITH

**Fig. 5.**
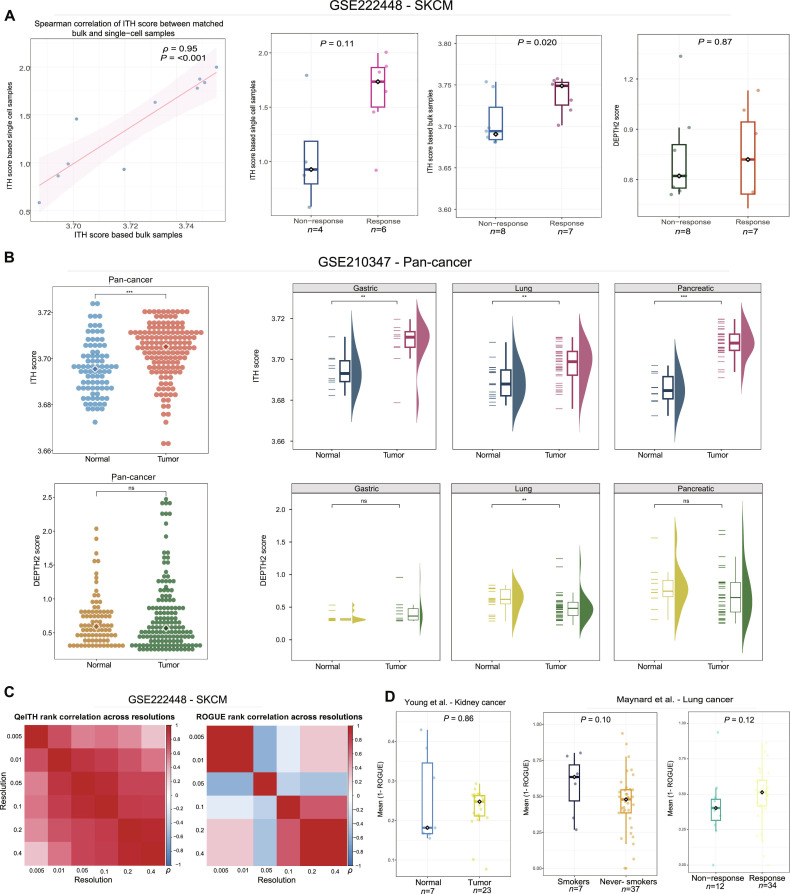
Robustness and benchmarking of QeITH. (A) Evaluation of QeITH performance in distinguishing immunotherapy responders from nonresponders using matched SKCM bulk and single-cell data. Left: Scatterplot showing strong Spearman correlation between ITH scores derived from bulk RNA-seq and cell composition-based ITH scores from single-cell data. Right: Boxplots with jittered points showing QeITH and DEPTH2 scores in responders versus nonresponders. (B) Pan-cancer comparison of QeITH and DEPTH2 scores between tumor and normal tissues in pseudobulk samples derived from scRNA-seq data. ITH scores were significantly elevated in tumors compared to normal tissues in pan-cancer analysis and validated in lung, pancreatic, and gastric cancers individually. DEPTH2 failed to detect significant tumor-normal differences in pan-cancer, pancreatic, or gastric cancer, and unexpectedly showed higher scores in normal lung tissues compared to tumors. Dot plots show individual sample values; each dot represents one sample. White diamonds indicate median values for each group. For the remaining panels, half-violins (right side) show density distributions; boxplots with overlaid line segments show median, IQR, and individual sample values (each line segment represents a single sample). (C) Stability of QeITH and ROGUE across clustering resolutions in SKCM single-cell data. Heatmaps showing Spearman rank correlations of sample rankings between resolution pairs. QeITH demonstrated high stability across resolutions, while ROGUE-based heterogeneity scores showed substantially lower consistency. (D) Comparison of QeITH and ROGUE in 2 independent single-cell datasets. Boxplots with jittered points show median, IQR, and individual sample values. Left: Kidney cancer dataset (Young et al.). QeITH detected significant differences between tumor and normal tissues, while ROGUE showed no significant difference. Right: Lung cancer dataset (Maynard et al.). QeITH showed trends approaching significance for smoker versus nonsmoker and responder versus nonresponder, while ROGUE showed no significant differences. The 2-tailed Mann–Whitney *U* test *P* values are shown.

To provide an initial assessment of QeITH’s performance across data modalities, we analyzed an SKCM cohort [45] with matched single-cell and bulk RNA-seq data. ITH scores derived from real bulk transcriptomic data showed strong concordance with cell composition-based ITH score calculated from single-cell data (Spearman *ρ* = 0.95, *P* < 0.001; Fig. 5A). When evaluating the ability to distinguish immunotherapy responders from nonresponders, QeITH scores from bulk data demonstrated significant predictive ability (*P* = 0.020; Fig. 5A). In the paired single-cell data, the cell composition-based ITH scores showed a weaker association that did not reach statistical significance (*P* = 0.114), although we note the limited sample size of the nonresponder group. We further benchmarked QeITH against DEPTH2, a transcriptomics-based method for quantifying heterogeneity, using the same matched SKCM bulk RNA-seq data. Notably, QeITH scores showed significant differences between immunotherapy responders and nonresponders (*P* = 0.020; Fig. 5A), whereas DEPTH2 scores showed no significant difference (*P* = 0.87; Fig. 5A).

We next extended our analysis to a pan-cancer pseudobulk dataset generated from scRNA-seq data [33], in which gene expression values for each simulated sample were calculated as the sum of counts across all constituent cells. ITH scores were significantly elevated in tumor samples compared to normal tissues (*P* = 0.03; Fig. 5B). This finding was validated in 3 individual cancer types with the largest sample sizes (lung, pancreatic, and gastric cancer), where tumor tissues uniformly showed significantly higher QeITH scores than normal counterparts (*P* < 0.05; Fig. 5B). In contrast, DEPTH2 failed to detect significant tumor-normal differences in the pan-cancer analysis, pancreatic cancer, or gastric cancer, and unexpectedly displayed higher scores in normal lung tissues compared to tumor tissues (*P* < 0.05; Fig. 5B).

To assess the sensitivity of QeITH to clustering parameters, we performed clustering on this SKCM single-cell dataset across 6 resolutions (0.005, 0.01, 0.05, 0.1, 0.2, and 0.4) and calculated ITH values for each sample at each resolution. As expected, QeITH values increased with higher resolutions (Fig. S4A). Critically, the relative ranking of samples remained highly stable across resolutions, with a mean Spearman correlation of 0.72 between different resolution pairs (Fig. 5C). This indicates that while absolute QeITH values are influenced by resolution, comparative conclusions based on sample rankings are robust to clustering parameter choices. We next compared QeITH with ROGUE; sample-level heterogeneity scores were calculated as the mean of (1-ROGUE) across all clusters within each sample. Across resolutions, ROGUE values decreased with increasing resolution and showed substantially lower consistency (mean rank correlation = 0.11) compared to QeITH (0.72), indicating that ROGUE is more sensitive to clustering resolution (Fig. 5C and Fig. S4B). Furthermore, the correlations between QeITH scores and ROGUE-based heterogeneity scores varied across resolutions, suggesting that the 2 metrics capture distinct aspects of transcriptional heterogeneity that are differentially influenced by clustering granularity (Fig. S4C).

We further applied ROGUE to 2 independent single-cell datasets. In the Young et al. kidney cancer dataset, ROGUE-based heterogeneity showed no significant difference between tumor and normal tissues (*P* = 0.86; Fig. 5D). In the Maynard et al. lung cancer dataset, no significant differences were observed between smokers versus nonsmokers or between treatment responders versus nonresponders (*P* > 0.10; Fig. 5D). In contrast, QeITH detected significant tumor-normal differences in the kidney cancer dataset (*P* < 0.05) and showed trends approaching significance in the lung cancer dataset (*P* = 0.052 and *P* = 0.059). These findings suggest that QeITH offers complementary sensitivity to ROGUE for detecting clinically relevant heterogeneity, reflecting their distinct focuses on intercluster versus intra-cluster diversity.

### ITH associates with unfavorable clinical outcomes in bulk tumors

**Fig. 6.**
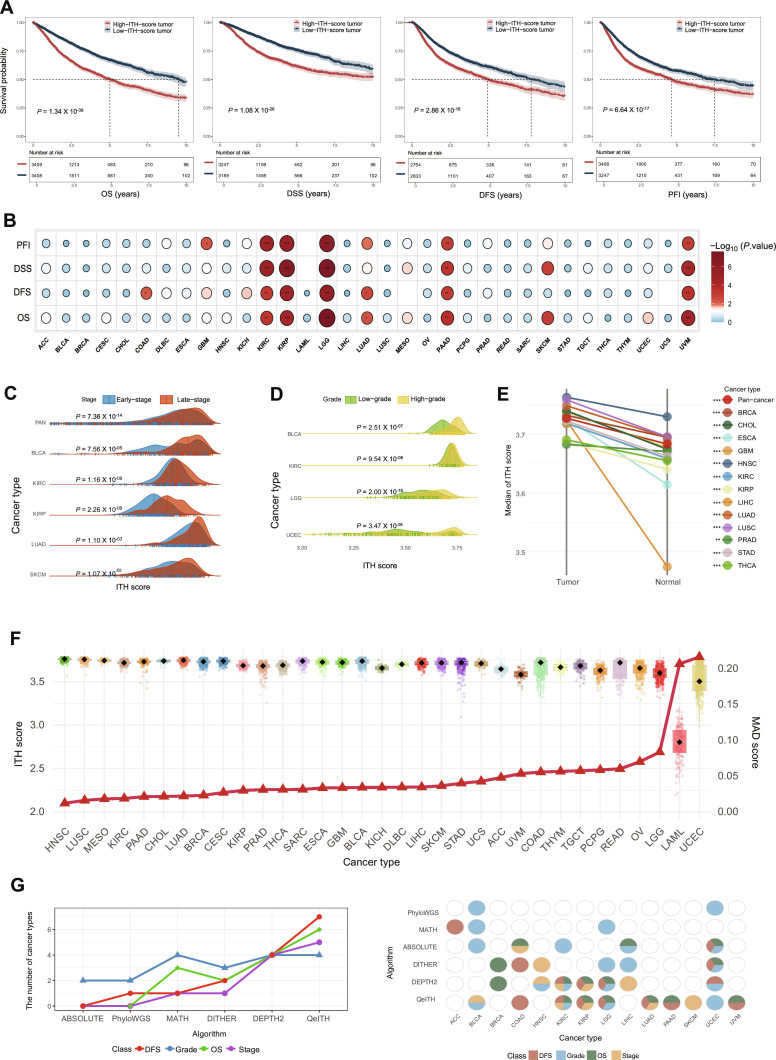
ITH scores are associated with unfavorable clinical outcomes in bulk tumors. (A) KM curves to compare the survival rates between the high-ITH-score (top third) and low-ITH-score (bottom third) patients in TCGA pan-cancer. The log-rank test *P* values are shown. OS, overall survival; DFS, disease-free survival; DSS, disease-specific survival; PFI, progression-free interval. (B) The ITH scores were significantly associated with survival outcomes at the individual cancer type level. The bubble chart presents *P* values for each cancer type. Cancer types with FDR < 0.05 are indicated by asterisks. Point size and color represents −log_10_(*P* value). (C and D) Ridge plots showing ITH score distributions stratified by tumor stage (C) and tumor grade (D). For each cancer type, density curves represent ITH distributions for early/late-stage or low/high-grade tumors; vertical lines indicate medians; overlaid line segments show individual sample values (each line = one sample). ITH scores were significantly lower in early-stage (stage I–II) than in late-stage (stage III–IV) tumors (C) and in low-grade (G1–2) than in high-grade (G3–4) tumors (D). (E) ITH scores were significantly higher in tumor than in normal samples. Slope graph showing median ITH score changes from tumor to normal samples across multiple cancer types; points indicate median values. (F) Comparison of the ITH scores and the MAD of ITH scores across 33 TCGA cancer types. Boxplots with jittered points show ITH score distributions for each cancer type (left axis); boxes indicate median and IQR; points represent individual samples. The line plot (right axis) shows MAD values, reflecting ITH variability within each cancer type. (G) Comparisons of QeITH with the other 5 ITH evaluation algorithms across cancer types. Left: Line plot showing the number of cancer types in which each algorithm demonstrated significant associations (FDR < 0.05) with clinical or pathological features (OS, stage, grade, and DFS). Methods are ordered by the total number of significant associations across all 4 metrics. Each line represents a different clinical/pathological feature, allowing comparison of algorithm performance across benchmarks. Right: Matrix of pie charts showing algorithm performance at the individual cancer type level. Each row represents an algorithm; each column represents a cancer type. Pie charts are color-coded to indicate which clinical/pathological features (OS, DFS, stage, grade) showed significant associations (FDR < 0.05) for each algorithm–cancer type pair. This visualization enables assessment of whether multiple methods converge on the same subset of cancer types. The 2-tailed Mann–Whitney *U* test *P* values are shown.

We applied QeITH to derive ITH scores for tumor samples within TCGA pan-cancer cohort (33 distinct cancer types) (Table S3). Survival analysis revealed that patients with low ITH scores had significantly better clinical outcomes than those in the high ITH scores group. This association was consistent across multiple survival endpoints—overall survival (OS), disease-specific survival (DSS), disease-free survival (DFS), and progression-free interval (PFI)—(log-rank test, *P* < 1.39 × 10^−6^; Fig. 6A). At the individual cancer type level, elevated ITH scores were significantly associated with adverse outcomes in 6, 7, 5, and 6 cancer types for OS, DSS, DFS, and PFI, respectively (FDR < 0.05; Fig. 6B and Fig. S5A). In addition, ITH scores were significantly lower in early stage (stage I–II) compared to late stage (stage III–IV) in the pan-cancer cohort and in 5 individual cancer types (FDR < 0.05; Fig. 6C). Likewise, ITH scores were significantly elevated in high-grade (G3–4) relative to low-grade (G1–2) tumors across 4 cancer types (FDR < 0.05; Fig. 6D). ITH scores were also significantly higher in tumor samples compared to matched normal tissues across pan-cancer and in 13 individual cancer types (FDR < 0.05; Fig. 6E).

To determine whether QeITH provides independent prognostic information beyond established factors, we constructed sequential multivariable Cox models for OS. The first model included stage and TMB. The second model further incorporated tumor purity and immune score; however, this addition did not significantly improve model fit (LRT: χ^2^ = 0.58, *P* = 0.750). Notably, the subsequent addition of QeITH in the third model resulted in a substantial improvement in model fit (LRT: χ^2^ = 178.92, *P* < 0.001). A simplified model including only stage, TMB, and QeITH also demonstrated significant improvement over the baseline model (LRT: χ^2^ = 136.42, *P* < 0.001). The magnitude of these improvements confirms that QeITH captures unique prognostic information independent of and incremental to stage, TMB, tumor purity, and immune score. In the fully adjusted model, QeITH remained significantly associated with OS [hazard ratio (HR) = 1.48, 95% confidence interval (CI): 1.39 to 1.57, *P* = 4.32 × 10^−36^; Fig. S5B]. To further validate these findings at the individual cancer type level, we performed cancer type-specific multivariate Cox analyses for OS. After adjusting for these covariates, ITH scores remained independently prognostic in 9 cancer types (FDR < 0.05; Fig. S5B). Among the 6 cancer types with significant OS differences in log-rank analysis, all remained significant after multivariable adjustment. To assess the robustness of our findings to the violation of proportional hazards assumption for stage (Schoenfeld test: *P* < 0.001), we performed sensitivity analyses using stage-stratified models. The results were highly consistent with the primary analysis: The addition of QeITH significantly improved model fit (LRT: χ^2^ = 180.69, df = 1, *P* < 0.001), and QeITH remained significantly associated with OS (HR = 1.48, 95% CI: 1.39 to 1.57, *P* = 2.19 × 10^−36^) in the fully adjusted stratified model, confirming the robustness of our conclusions (Fig. S5C).

We computed the MAD of ITH scores to assess intra-cancer type variability (Fig. 6F). We stratified all cancer types into 4 groups based on the median values of MAD and median ITH: group 1 (low MAD + low ITH), group 2 (high MAD + low ITH), group 3 (low MAD + high ITH), and group 4 (high MAD + high ITH). Each patient was then assigned to their corresponding cancer type group for KM survival analysis. Patients in group 3, which includes cancers with low MAD and high ITH (HNSC, LUSC, and MESO), exhibited the poorest prognosis among all groups (log-rank test, *P* < 0.001; Fig. S5D). This finding indicates that the combination of low intra-cancer type variability and high ITH is associated with aggressive malignancies, suggesting that stable, high ITH profiles may characterize tumors with poorer clinical outcomes.

We benchmarked QeITH against 5 established ITH estimation algorithms (MATH, PhyloWGS, ABSOLUTE, DEPTH2, and DITHER) across the 33 TCGA cohorts. QeITH demonstrated more robust associations with clinical outcomes: QeITH was negatively correlated with OS in 6 cancer types, outperforming MATH (5 types), PhyloWGS (0), ABSOLUTE (0), DEPTH2 (4), and DITHER (2) (FDR < 0.05) (Fig. 6G and Fig. S5E). In addition, QeITH was associated with shorter DFS in 7 cancer types, exceeding the performance of most other methods, including MATH (1 type), PhyloWGS (1), ABSOLUTE (0), DEPTH2 (4), and DITHER (2) (FDR < 0.05; Fig. 6G and Fig. S5E). QeITH revealed significant differences in both stage (early stage versus late stage) and grade (low grade versus high grade). For stage, QeITH identified 5 cancer types, while MATH identified 1 type, PhyloWGS identified 0 types, ABSOLUTE identified 0 types, DEPTH2 identified 4 types, and DITHER identified 1 type (FDR < 0.05). In terms of grade, QeITH identified 4 types, matching or surpassing the performance of MATH (4 types), PhyloWGS (2), ABSOLUTE (2), DEPTH2 (4), and DITHER (3) (FDR < 0.05; Fig. 6G and Fig. S5E). These results indicate that the QeITH algorithm demonstrates a more robust association with unfavorable clinical outcomes in cancer compared to existing ITH algorithms.

To assess whether QeITH reflects distinct biological dimensions compared to genomic ITH metrics, we performed Spearman correlation analyses between QeITH and 2 genomic ITH metrics (MATH and PhyloWGS) across 33 TCGA cancer types. We observed significant correlations in 7 and 11 cancer types for MATH and PhyloWGS, respectively (FDR < 0.05; Fig. S5F). The correlations were modest (max *ρ* = 0.42 for MATH in LAML; 0.49 for PhyloWGS in THYM), with both positive and negative associations observed across cancer types. Importantly, the majority of cancer types showed no significant correlation with either metric, indicating that QeITH captures ecosystem-level features largely orthogonal to genetic subclonal diversity. These findings empirically support QeITH as an ecosystem heterogeneity index that complements, rather than duplicates, classical genomic ITH metrics.

We further assessed the robustness of QeITH to methodological choices. The global shift method for handling negative values correlated highly with the original zero-thresholding approach (*ρ* = 0.976, *P* < 0.001; Fig. S5G). Alternative normalization schemes showed correlations with the original method (rank-based quantile normalization: *ρ* = 0.862, *P* < 0.001; square normalization: *ρ* = 0.934, *P* < 0.001; Fig. S5G). To assess robustness to gene set composition, we performed random subsampling of 6 to 13 gene sets from the original 14. Notably, even when using only 10 gene sets (removing 4 states), the mean correlation with the original ITH scores remained 0.922 (*P* < 0.001), indicating that QeITH captures a global property of the TME that is largely robust to specific gene set composition (Fig. S5G).

### ITH correlates with unfavorable therapy response and molecular features

**Fig. 7.**
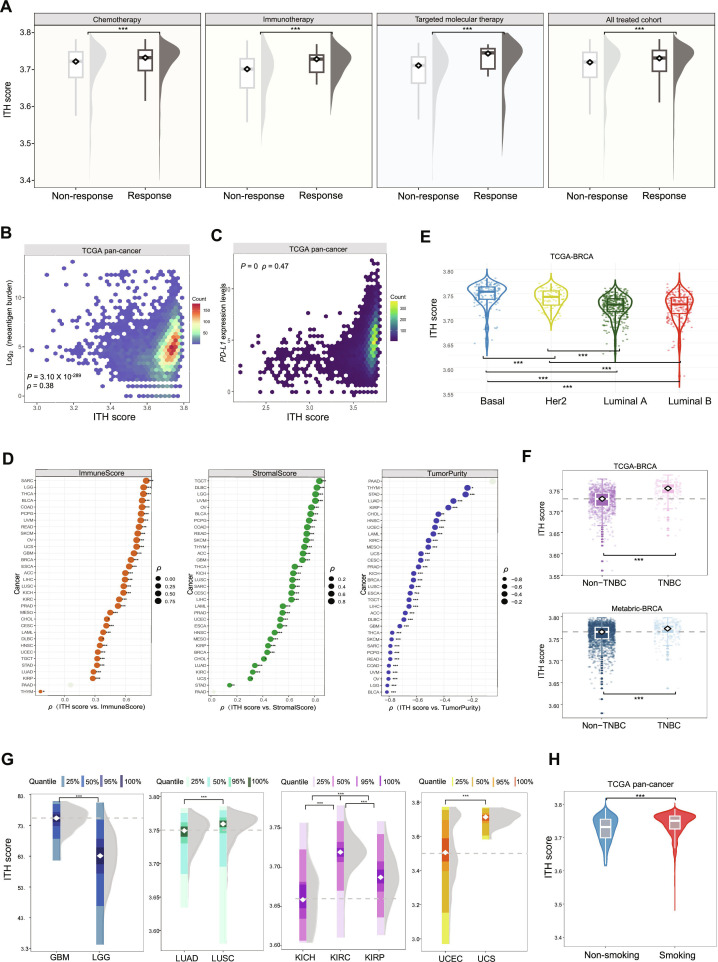
ITH scores correlate with favorable therapy response and molecular features in bulk tumors. (A) ITH scores were significantly higher in tumors that responded to therapy, with consistent patterns observed across multiple treatment approaches, including chemotherapy, targeted molecular therapy, and immunotherapy. Boxplots with overlaid jittered points show median, IQR, and individual sample values. Half-violins on the side show density distributions of ITH scores. (B and C) Hexbin plots showing correlations between ITH scores and tumor neoantigen burden (B) and *PD-L1* expression levels (C) in pan-cancer analyses. Each hexagon represents a region of the scatterplot; color intensity indicates the density of samples within that region. ITH scores showed significantly positive correlations with both neoantigen burden and *PD-L1* expression. (D) Dot plots showing Spearman correlations between ITH scores and immune score, stromal score, and tumor purity in TCGA pan-cancer and 33 individual cancer types. Each dot represents the correlation estimate (*ρ*) for a specific cancer type; dot size reflects the magnitude of the correlation. Cancer types with FDR < 0.05 are indicated by asterisks. (E) Violin plots showing ITH score distributions across breast cancer subtypes (luminal A, luminal B, HER2-enriched, and basal-like) in TCGA-BRCA. Violins show density distributions; boxplots indicate median and IQR; jittered points represent individual sample value. ITH scores progressively increased with tumor aggressiveness: basal-like > HER2-enriched > luminal B > luminal A. (F) Boxplots with overlaid jittered points showing ITH score comparisons between TNBC and non-TNBC in TCGA-BRCA and Metabric-BRCA cohorts. Boxplots indicate median and IQR; jittered points represent individual sample value. (G) The cancers originating in the same tissue or organ but from distinct cells or parts show noticeably distinct ITH scores. Density curves show the distribution of ITH scores; colored interval bars represent percentile ranges (25%, 50%, 95%, and 100%); white diamonds mark median values. (H) Violin plots with overlaid boxplots show ITH score distributions in smokers versus never-smokers in the TCGA pan-cancer cohort. Patients with smoking history demonstrated significantly higher ITH scores than never-smokers. The 2-tailed Mann–Whitney *U* test *P* values are shown in (A and E to G). The Spearman correlation coefficients (*ρ*) and *P* values are shown in (B) to (D).

We evaluated the association between ITH scores and clinical response to therapies within the TCGA pan-cancer dataset. ITH scores were significantly elevated in responders compared with nonresponders across the entire treatment cohort (*P* < 0.001; Fig. 7A). This pattern was consistently observed when analyzing specific treatment modalities, such as chemotherapy, targeted molecular therapy, and immunotherapy (*P* < 0.05; Fig. 7A). We further examined the predictive value of QeITH in 3 independent immunotherapy datasets. Consistently, higher ITH scores were significantly associated with improved response to immune checkpoint blockade in 2 SKCM cohorts (*P* = 0.024 [44]; *P* = 0.028 [43]) and 1 NSCLC cohort (*P* = 0.038; Fig. S6A). To evaluate the incremental predictive value of QeITH beyond existing biomarkers, multivariable logistic regression analyses were performed in TCGA pan-cancer . When added to a baseline model containing *PD-L1* expression, QeITH remained a significant independent predictor (coefficient = 0.112, *P* = 0.044), and the LRT confirmed significant improvement in model fit (*P* = 0.044). These results indicate that QeITH provides incremental predictive value beyond *PD-L1* expression .

We also assessed the association between ITH scores and molecular features in TCGA cohorts (Table S4). A significant positive correlation was observed between ITH scores and tumor neoantigen burden in pan-cancer (*P* = 3.10 × 10^−289^, *ρ* = 0.38; Fig. 7B) and in 11 individual cancer types (*P* < 0.05; Fig. S6B). In the immune profile, ITH scores showed a strong positive association with *PD-L1* expression levels in pan-cancer (*P* < 0.001, *ρ* = 0.47; Fig. 7C) and in 31 individual cancer types (*P* < 0.05; Fig. S6C). ITH scores were positively correlated with immune and stromal scores in the pan-cancer cohort (*P* < 0.001) and most individual cancer types (FDR < 0.05), whereas they were inversely correlated with tumor purity (FDR < 0.05; Fig. 7D).

Furthermore, we compared ITH scores between different cancer subtypes, and found ITH scores to be elevated in more aggressive cancer subtypes. In TCGA-BRCA, ITH scores followed the pattern: basal-like > HER2-enriched > luminal B > luminal A (Kruskal–Wallis test, *P* < 0.001; Fig. 7E). TNBC was significantly higher than non-TNBC in both TCGA-BRCA (*P* = 7.49 × 10^−38^) and Metabric-BRCA cohorts (*P* = 6.77 × 10^−19^; Fig. 7F). In gliomas, the aggressive GBM showed significantly higher ITH scores than LGG (*P* = 3.89 × 10^−59^; Fig. 7G). For tissue-specific differences, LUAD displayed lower ITH scores than LUSC (*P* = 2.08 × 10^−16^; Fig. 7G). Among kidney cancers, the order of ITH scores was KIRC > KIRP > KICH (*P* = 2.0 × 10^−66^; Fig. 7G). Additionally, smoking, which is a major cancer risk factor, was associated with elevated ITH scores in the pan-cancer cohort (*P* = 1.72 × 10^−29^; Fig. 7H)

### Spatial transcriptomics analysis reveals increased ITH scores in invasive regions

**Fig. 8.**
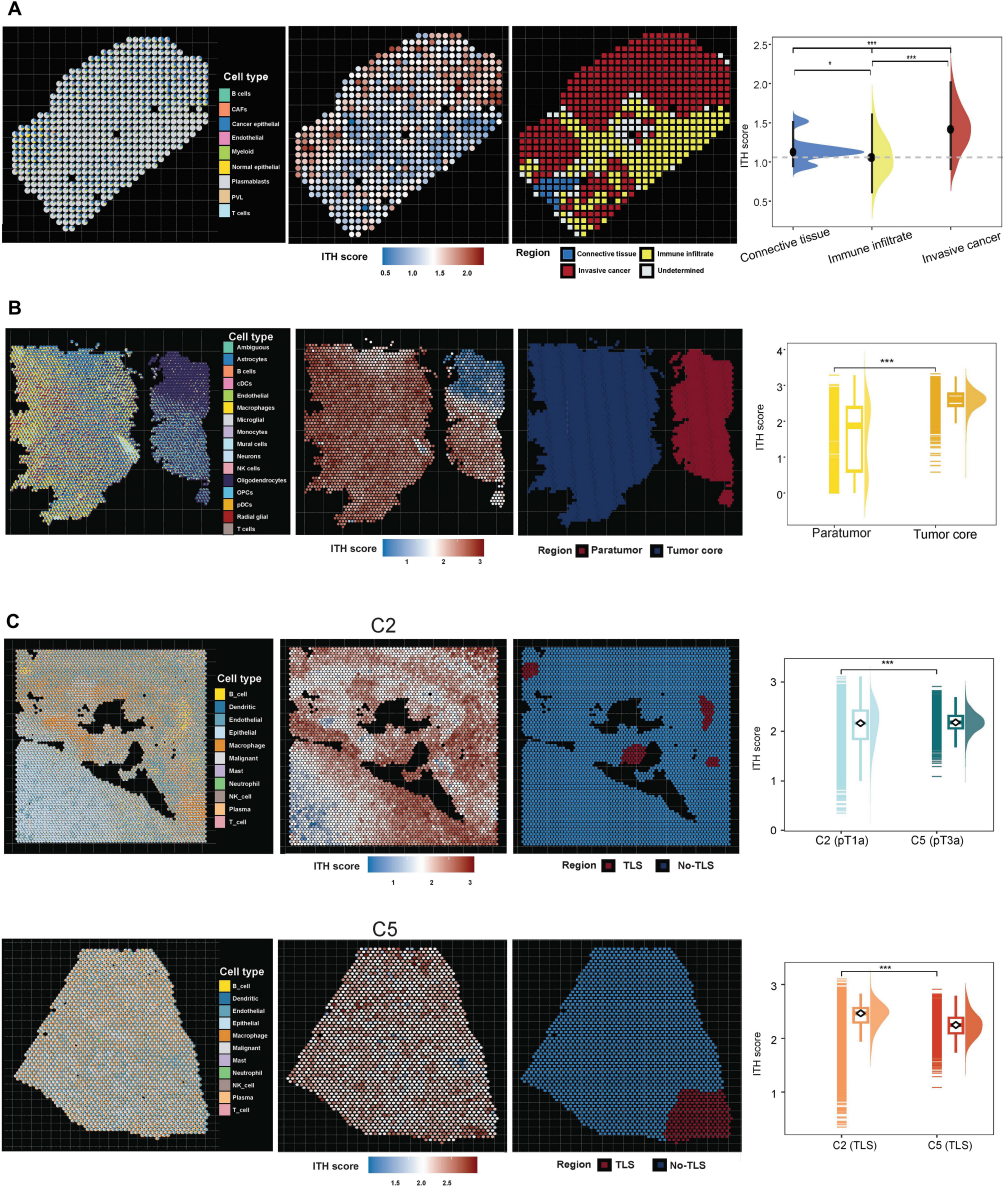
Spatial transcriptomic analysis reveals increased ITH scores in invasive regions. (A) Spatial transcriptomics analysis of a BRCA sample. Left: Cell type composition determined by CARD deconvolution, showing the proportion of each cell type per spot. Center: Spatial mapping of ITH scores across the tissue section. Right: H&E-stained tissue section with 4 annotated regions based on morphological features. Half-eye plots show ITH score distributions across regions (slabs = density; interval bars = 10% and 95% credible intervals; points = medians). ITH scores were significantly higher in invasive cancer regions compared to other regions. (B) Glioblastoma spatial transcriptomics. Left: Cell type annotation across tissue sections using paired spatial transcriptomic and single-cell data from the same patients. Center: Spatial mapping of ITH scores across the tissue section. Right: Paratumor and tumor core regions. Raincloud plots show ITH score distributions: Half-violins (right side) show density distributions; boxplots indicate median and IQR; overlaid line segments represent individual spot values (each line = one spot)*.* ITH scores were significantly higher in the tumor core than in the paratumor region. (C) RCC spatial transcriptomics. Left: Cell type composition determined by CARD deconvolution. Center: Spatial mapping of ITH scores across tissue sections with TLS and non-TLS regions annotated. Right: Annotation of TLS and non-TLS regions. Raincloud plots comparing ITH scores (top: comparing overall ITH scores between C2 and C5 samples; bottom: comparing ITH scores within TLS regions between C2 and C5). Half-violins show density distributions; boxplots indicate median and IQR; line segments represent individual spot values (each line = one spot). C5 showed higher ITH scores than C2 (top), while C2 exhibited significantly higher ITH scores than C5 within TLS regions (bottom). The Kruskal–Wallis test *P* value is shown in (A), and 2-tailed Mann–Whitney *U* test *P* values are presented in (A) to (C).

Growing evidence indicates that the spatial configuration of the TME not only governs ITH but also provides a rich source of prognostic biomarkers [55]. To systematically evaluate the association between ITH levels and spatial context, we analyzed a BRCA spatial transcriptomic dataset [47] alongside a paired scRNA-seq dataset [24]. We applied the CARD algorithm to deconvolute cellular composition from the spatial profiles and subsequently computed spot-level ITH scores using the QeITH framework (Fig. 8A). Pathologist annotations of hematoxylin and eosin (H&E)-stained sections identified 4 distinct tissue regions (e.g., connective tissue, immune infiltrate, and invasive cancer) (Fig. 8A). Spatial ITH scores varied significantly across these regions (Kruskal–Wallis test, *P* = 3.46 × 10^−6^), with invasive cancer regions exhibiting markedly elevated ITH compared to all other tissue types (Mann–Whitney *U* test, *P* < 0.01; Fig. 8A). Spatial autocorrelation analysis confirmed nonrandom spatial organization of ITH (Moran’s *I* = 0.420, *P* < 0.0001), validating that high ITH areas form distinct spatial clusters within invasive regions. The mean ITH score followed the pattern: invasive cancer > connective tissue > immune infiltrate. We validated this spatial pattern in an independent 10x Genomics Xenium BRCA dataset [24], where invasive tumor regions infiltrated by lymphocytes again showed significantly higher ITH scores than noninvasive ductal carcinoma in situ (DCIS1) regions (*P* = 6.0 × 10^−4^; Fig. S4A).

This spatial pattern of ITH elevation was further observed across anatomically distinct samples from other cancer types. In a glioblastoma cohort [48], tumor core regions displayed significantly higher ITH scores compared to paratumor regions (*P* < 0.05; Fig. 8B). In pancreatic cancer [49], metastatic lesions exhibited elevated ITH relative to primary tumor regions (*P* < 0.05; Fig. S4B). Furthermore, in renal cell cancer (RCC) [50], the presence of intratumoral tertiary lymphoid structures (TLSs) is associated with improved responses to immunotherapy [50]. In a comparative analysis of 2 TLS-positive samples, the more aggressive pT3a sample (C5) exhibited a significantly higher ITH score than the pT1a sample (C2) (*P* < 0.05). Interestingly, within the TLS regions, the C5 sample demonstrated a lower ITH score, while the C2 sample showed a higher ITH score and a better immunotherapy response (*P* < 0.05; Fig. 8C). Spatial autocorrelation analysis revealed markedly different ITH organization patterns between the 2 RCC samples. The early-stage pT1a sample (C2) exhibited strong spatial clustering (Moran’s *I* = 0.694, *P* < 0.0001), while the aggressive pT3a sample (C5) showed substantially weaker spatial autocorrelation (Moran’s *I* = 0.168, *P* < 0.0001), indicating disrupted spatial architecture in advanced tumors. This finding is consistent with previous analyses, which indicate that higher ITH scores might be associated with better treatment responses.

Collectively, these multi-cancer spatial analyses establish that ITH is consistently elevated in anatomically defined invasive and metastatic tumor regions. This pattern suggests that localized increases in microenvironmental diversity are closely linked to tumor aggressiveness and progression, while the heterogeneity within specific immune structures may harbor additional prognostic significance.

## Discussion

ITH is a recognized driver of malignant progression and therapeutic failure [2,56]. While existing methods primarily focus on genetic (mutational) heterogeneity, our work introduces QeITH, a novel entropy-based metric designed to quantify ITH by capturing the ecological complexity of the entire TME. By leveraging transcriptomics to assess the diversity and distribution of cellular populations and associated functional states, QeITH overcomes the limitations of genomic methods, which often overlook the TME’s critical role in shaping tumor evolution. The principal innovation of QeITH lies in its reconceptualization of ITH. This paradigm shifts from a cancer-centric to an ecosystem-centric view, allowing QeITH to directly quantify the diversity and distribution of all cell populations or functional states within the TME. This is most accurately realized in scRNA-seq data, where QeITH operates on the precise proportions of annotated cell types. When applied to bulk transcriptomic data, the algorithm infers the relative abundances of 14 fundamental tumor-associated functional states, providing a proxy for the TME’s cellular heterogeneity.

The biological relevance of QeITH is strongly supported by its correlation with key molecular features. We demonstrated that QeITH scores are positively associated with immune and stromal scores and inversely correlated with tumor purity. This validates that QeITH successfully captures TME features rather than just the cancer cell fraction, positioning it as an ecological metric. Furthermore, the association of high ITH with elevated *PD-L1* expression and neoantigen burden confirms that QeITH quantifies a biologically active, high-TMB-like immune ecosystem highly pertinent to modern cancer therapy.

QeITH scores consistently and robustly distinguished malignant tissue from normal tissues across pan-cancer and individual cohorts, establishing its fundamental role in distinguishing malignant transformation. Crucially, QeITH scores increased proportionally with advancing tumor stage and grade, reflecting the established concept that greater ecological complexity fuels tumor aggressiveness. This pattern was reinforced by the association of high ITH with aggressive subtypes, such as TNBC (basal-like) and KIRC.

When benchmarking against established ITH algorithms (e.g., MATH, ABSOLUTE, PhyloWGS, DEPTH2, and DITHER) across TCGA cohorts, the TME-centric QeITH metric demonstrated associations with clinical outcomes (OS, DFS, stage, and grade) in a larger number of cancer types. However, these comparisons should not be interpreted as demonstrating superiority, as each metric captures a distinct dimension of heterogeneity: MATH (mutant-allele), ABSOLUTE/PhyloWGS (clonal architecture), DEPTH2 (transcriptome levels), DITHER (genomic alterations), and QeITH (ecosystem heterogeneity). The observed differences are therefore expected and reflect the multi-faceted nature of intratumoral heterogeneity. Beyond bulk tissue analysis, we extended our evaluation to single-cell datasets using ROGUE, a metric designed to quantify cluster purity where 1-ROGUE reflects within-cluster transcriptional heterogeneity—a dimension not captured by QeITH. Conversely, ROGUE does not account for the compositional diversity arising from the distribution of multiple clusters within a sample, which is precisely what QeITH quantifies through population entropy. More broadly, each existing method offers unique advantages: MATH and DITHER provide insight into genomic instability; ABSOLUTE and PhyloWGS reconstruct clonal evolution; DEPTH2 offers a malignancy-focused transcriptional perspective; and ROGUE assesses cluster purity. The choice of method should therefore be guided by the specific biological question, data availability, and desired level of resolution.

A critical finding is the paradoxical dual role of high ITH in clinical outcomes. High ITH is associated with unfavorable long-term prognosis (shorter OS, DFS) across multiple TCGA cohorts. This likely reflects the detrimental effect of high ecosystem diversity, where a large repertoire of cell states (phenotypic plasticity) allows the tumor to resist microenvironmental pressures and treatment. Conversely, high ITH was markedlyassociated with a favorable response to multiple treatment modalities, including chemotherapy, targeted molecular therapy, and immunotherapy. This seemingly contradictory finding is consistent with the metric’s TME focus. High ITH, characterized by high immune score and *PD-L1* expression, suggests the presence of a highly infiltrated, or “hot”, TME. Such tumors are known to respond better to immunotherapies and other treatments that rely on active host–tumor interaction. Thus, QeITH may serve as a predictive biomarker identifying patients who would most benefit from aggressive therapeutic intervention.

Additionally, the application of QeITH to spatial transcriptomics data provides novel insight into the spatial evolutionary dynamics of ITH. We found that ITH scores were consistently elevated in invasive cancer regions and metastatic lesions compared to primary or normal tissue. This spatial pattern validates the theory that the tumor–host interface is a key site of local evolutionary branching and microenvironmental remodeling. The variability observed within specific TLS regions also suggests that quantifying ITH in subregions of the TME holds additional prognostic information.

QeITH extends previous entropy-based approaches by introducing an ecosystem-level perspective that integrates multiple data modalities. While methods such as DITHER [7] quantify genomic heterogeneity, and other entropy frameworks have been applied to transcriptomic [11] and spatial [12] data, each captures only a single dimension of tumor complexity. QeITH differs fundamentally by measuring transcriptional population diversity from cluster compositions—reflecting ecological complexity across the entire TME—through a unified framework applicable to single-cell, bulk, and spatial transcriptomic data. In contrast, single-cell-specific metrics like ROGUE focus on within-cluster purity (where 1-ROGUE represents intra-cluster heterogeneity), making them sensitive to clustering resolution and better suited for cluster quality assessment than cross-sample comparisons of overall heterogeneity.

Several limitations of QeITH should be acknowledged. First, the method is clustering-dependent, as cluster definitions directly impact entropy calculations, and within-cluster heterogeneity is not captured. Furthermore, QeITH does not capture genetic heterogeneity that may not manifest transcriptionally, which may exist as a transcriptionally silent reservoir for therapy-induced evolution. QeITH cannot distinguish between heterogeneity arising from malignant subclones versus infiltrating immune cells, limiting attribution of observed diversity to specific cellular compartments. Current analyses are based on static measurements, missing dynamic heterogeneity changes during treatment. An additional technical consideration is cross-dataset comparability. While QeITH scores are comparable within uniformly processed datasets, direct comparison of absolute ITH scores across different platforms, technologies, or annotation resolutions should be approached with caution, as technical factors can influence absolute ITH scores. Future improvements could address these limitations through clustering-free entropy measures, hierarchical frameworks capturing both inter- and intra-cluster heterogeneity, multi-omic integration to capture latent genetic heterogeneity, decomposition into cell type-specific entropy components, longitudinal entropy metrics from serial biopsies, and batch-effect correction methods to enhance cross-dataset comparability. Ultimately, prospective clinical validation will determine QeITH’s utility as a decision support tool.

In summary, we have developed QeITH, a robust and biologically relevant metric that successfully quantifies the ecological complexity of the tumor ecosystem across multiple modalities. While QeITH leverages the classical Shannon entropy measure, its methodological contribution lies in the integrated cross-modality implementation and systematic validation across multiple cancer types and data platforms. Our findings establish QeITH as a powerful biomarker for cancer prognosis, subtype stratification, and therapeutic prediction, providing a new dimension for understanding and combating ITH.

## Data Availability

All data associated with this study are available within the paper and its Supplementary Materials. The QeITH R package is publicly available for download and installation at the GitHub repository (https://github.com/XS-Wang-Lab/QeITH). The web application of QeITH is available at the website: https://xs-wang-lab.shinyapps.io/qeith-shiny.
