# Supplementary material for: QeITH: Quantifies Tumor Ecosystem Heterogeneity to Predict Cancer Progression and Treatment Benefit
Source: Comput Struct Biotechnol J. 2026 Jun 18;35(1):0061. doi: 10.34133/csbj.0061 (PMC13276245; doi:10.34133/csbj.0061)
Supplement: Supplementary 1 — Figs. S1 to S7 Tables S1 to S5 [file csbj.0061.f1.zip › FIG.S2.pdf]

Fig. S2

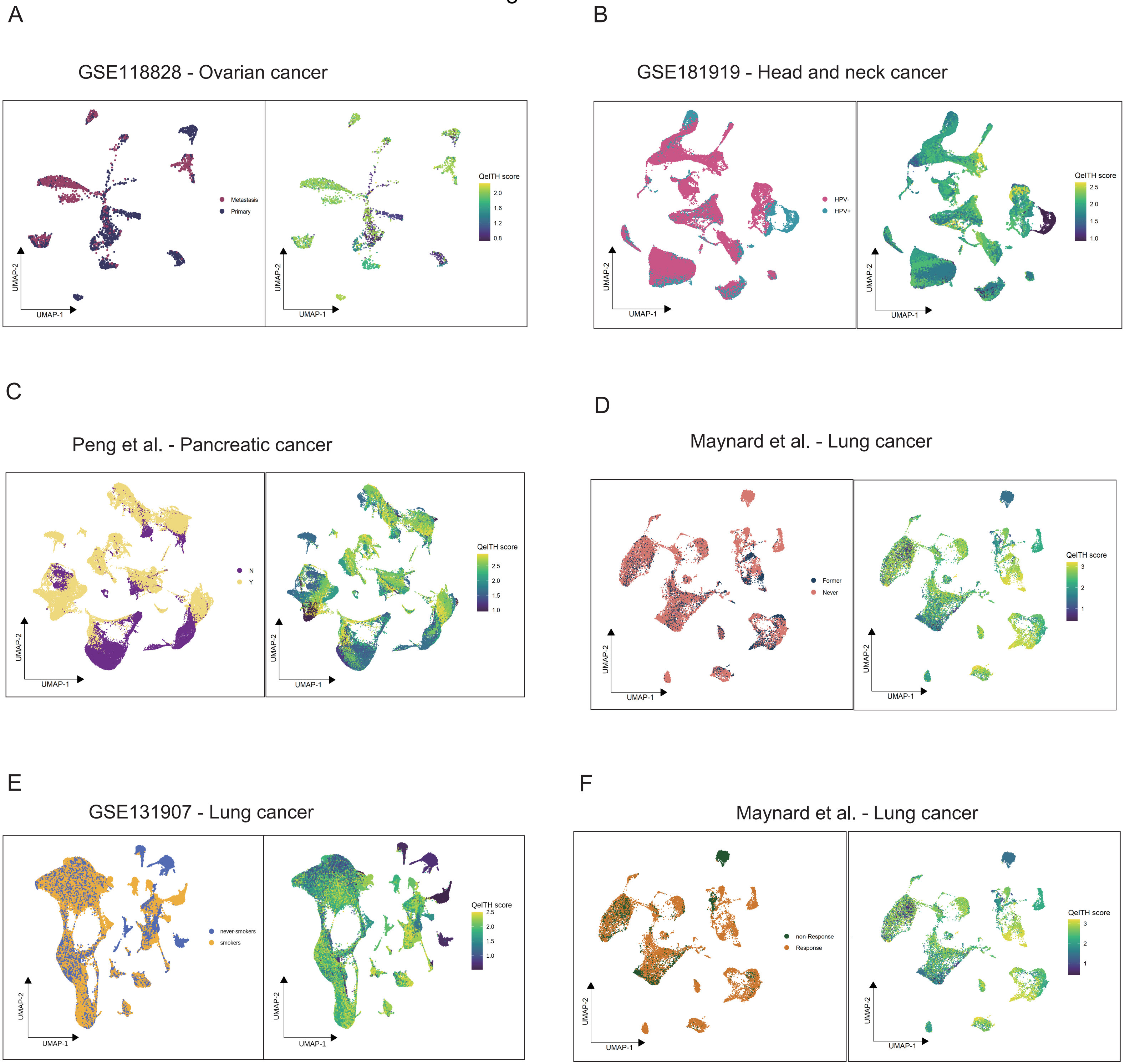
A
GSE118828 - Ovarian cancer
Metastasis
Primary
QeITH score
2.0
1.6
1.2
0.8
UMAP-1
UMAP-2
B
GSE181919 - Head and neck cancer
HPV-
HPV+
QeITH score
2.5
2.0
1.5
1.0
C
Peng et al. - Pancreatic cancer
N
Y
QeITH score
2.5
2.0
1.5
1.0
D
Maynard et al. - Lung cancer
Former
Never
QeITH score
3
2
1
E
GSE131907 - Lung cancer
never-smokers
smokers
QeITH score
2.5
2.0
1.5
1.0
F
Maynard et al. - Lung cancer
non-Response
Response
QeITH score
3
2
1
