# Supplementary material for: QeITH: Quantifies Tumor Ecosystem Heterogeneity to Predict Cancer Progression and Treatment Benefit
Source: Comput Struct Biotechnol J. 2026 Jun 18;35(1):0061. doi: 10.34133/csbj.0061 (PMC13276245; doi:10.34133/csbj.0061)
Supplement: Supplementary 1 — Figs. S1 to S7 Tables S1 to S5 [file csbj.0061.f1.zip › FIG.S3.pdf]

Fig. S3

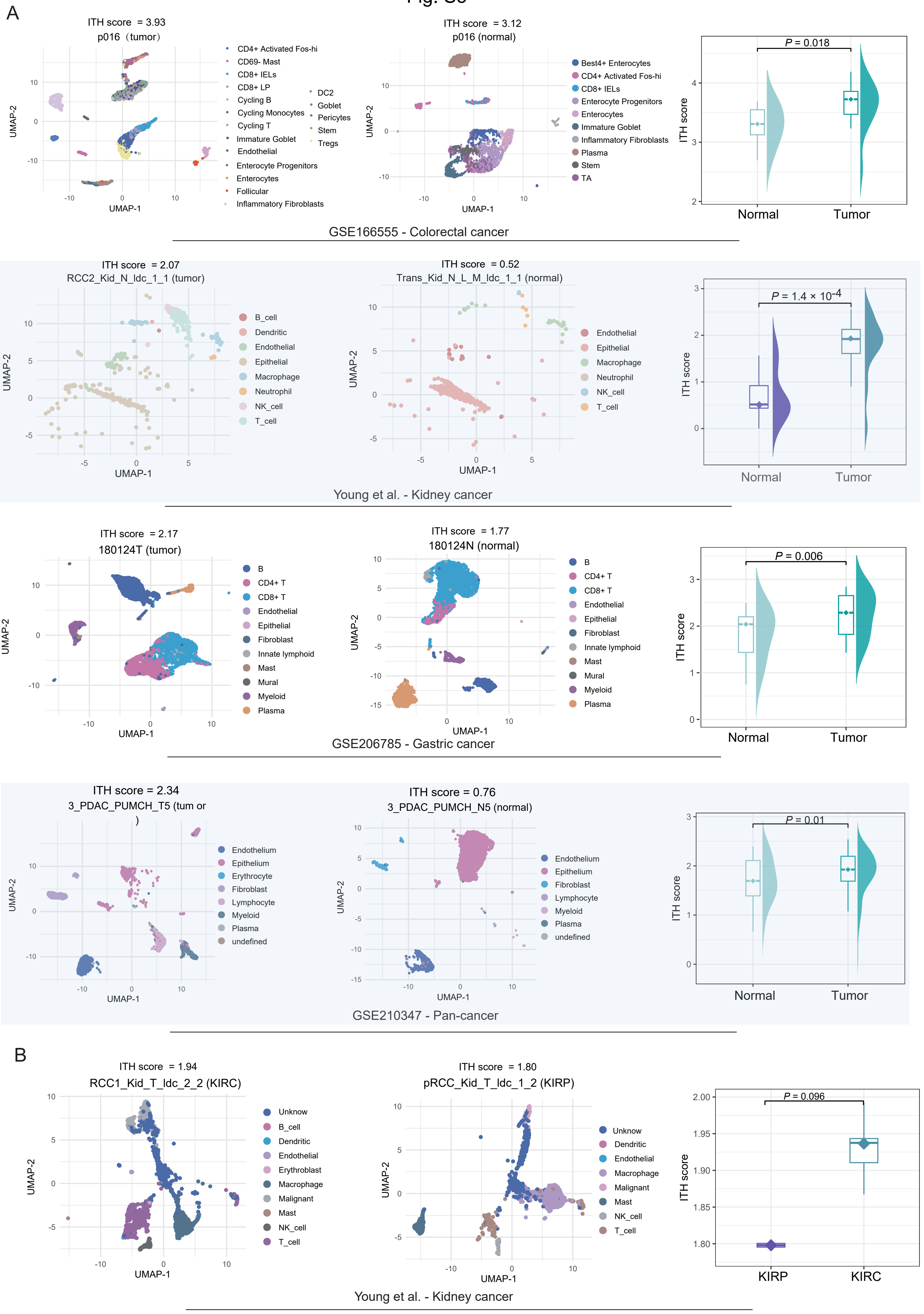
A
ITH score = 3.93
p016（tumor）
CD4+ Activated Fos-hi
CD69- Mast
CD8+ IELs
CD8+ LP
Cycling B
Cycling Monocytes
Cycling T
Immature Goblet
Endothelial
Enterocyte Progenitors
Enterocytes
Follicular
Inflammatory Fibroblasts
DC2
Goblet
Pericytes
Stem
Tregs
UMAP-1
UMAP-2
ITH score = 3.12
p016 (normal)
Best4+ Enterocytes
CD4+ Activated Fos-hi
CD8+ IELs
Enterocyte Progenitors
Enterocytes
Immature Goblet
Inflammatory Fibroblasts
Plasma
Stem
TA
P = 0.018
ITH score
Normal
Tumor
GSE166555 - Colorectal cancer
ITH score = 2.07
RCC2_Kid_N_ldc_1_1 (tumor)
B_cell
Dendritic
Endothelial
Epithelial
Macrophage
Neutrophil
NK_cell
T_cell
ITH score = 0.52
Trans_Kid_N_L_M_ldc_1_1 (normal)
Endothelial
Epithelial
Macrophage
Neutrophil
NK_cell
T_cell
P = 1.4 × 10-4
Young et al. - Kidney cancer
ITH score = 2.17
180124T (tumor)
B
CD4+ T
CD8+ T
Endothelial
Epithelial
Fibroblast
Innate lymphoid
Mast
Mural
Myeloid
Plasma
ITH score = 1.77
180124N (normal)
P = 0.006
GSE206785 - Gastric cancer
ITH score = 2.34
3_PDAC_PUMCH_T5 (tum or
)
Endothelium
Epithelium
Erythrocyte
Fibroblast
Lymphocyte
Myeloid
Plasma
undefined
ITH score = 0.76
3_PDAC_PUMCH_N5 (normal)
Endothelium
Epithelium
Fibroblast
Lymphocyte
Myeloid
Plasma
undefined
P = 0.01
GSE210347 - Pan-cancer
B
ITH score = 1.94
RCC1_Kid_T_ldc_2_2 (KIRC)
Unknow
B_cell
Dendritic
Endothelial
Erythroblast
Macrophage
Malignant
Mast
NK_cell
T_cell
ITH score = 1.80
pRCC_Kid_T_ldc_1_2 (KIRP)
Unknow
Dendritic
Endothelial
Macrophage
Malignant
Mast
NK_cell
T_cell
P = 0.096
KIRP
KIRC
Young et al. - Kidney cancer
