# Supplementary material for: QeITH: Quantifies Tumor Ecosystem Heterogeneity to Predict Cancer Progression and Treatment Benefit
Source: Comput Struct Biotechnol J. 2026 Jun 18;35(1):0061. doi: 10.34133/csbj.0061 (PMC13276245; doi:10.34133/csbj.0061)
Supplement: Supplementary 1 — Figs. S1 to S7 Tables S1 to S5 [file csbj.0061.f1.zip › FIG.S4.pdf]

Fig. S4

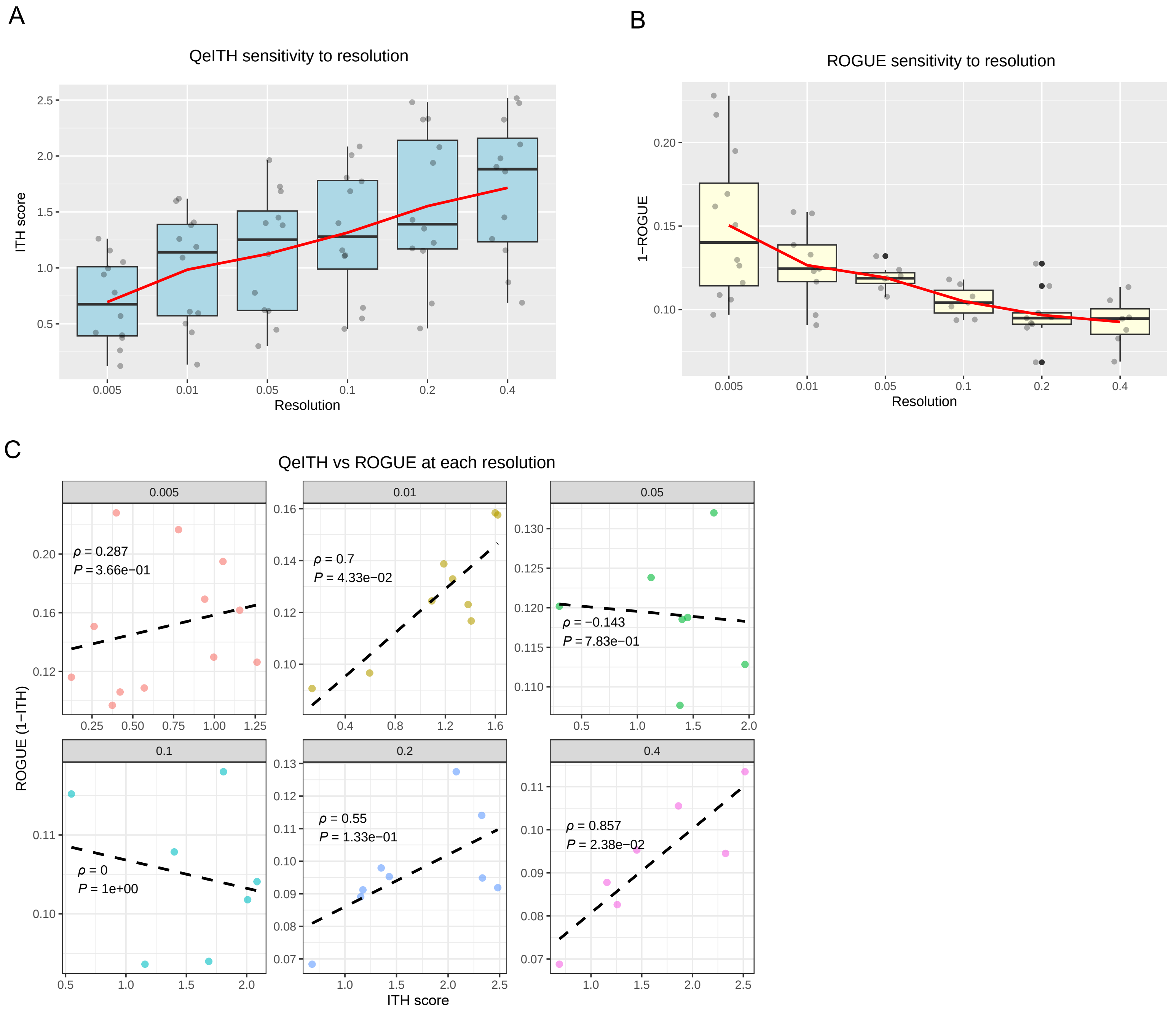
A
QeITH sensitivity to resolution
ITH score
Resolution
0.005
0.01
0.05
0.1
0.2
0.4
B
ROGUE sensitivity to resolution
1−ROGUE
Resolution
C
QeITH vs ROGUE at each resolution
ROGUE (1−ITH)
ITH score
0.005
ρ = 0.287
P = 3.66e−01
0.01
ρ = 0.7
P = 4.33e−02
0.05
ρ = −0.143
P = 7.83e−01
0.1
ρ = 0
P = 1e+00
0.2
ρ = 0.55
P = 1.33e−01
0.4
ρ = 0.857
P = 2.38e−02
