# Supplementary material for: QeITH: Quantifies Tumor Ecosystem Heterogeneity to Predict Cancer Progression and Treatment Benefit
Source: Comput Struct Biotechnol J. 2026 Jun 18;35(1):0061. doi: 10.34133/csbj.0061 (PMC13276245; doi:10.34133/csbj.0061)
Supplement: Supplementary 1 — Figs. S1 to S7 Tables S1 to S5 [file csbj.0061.f1.zip › Supplementary Materials.docx]

**Supplemental files**

**Supplemental Tables.**

**Table S1.** **Gene signatures for 14 tumor-associated functional states.**

**Table S2. Summary of the datasets.**

**Table S3. ITH scores derived from TCGA samples using the QeITH.**

**Table S4**. **Correlation analyses between ITH scores and molecular features across cancer types.**

**Table S5**. **Summary of R packages and parameters used in this study.**

**Supplemental Figures**

**Fig. S1. Elevated ITH scores are associated with tumor progression phenotypes in single-cell datasets. (A-B)** UMAP visualizations in breast cancer **(A)** and pancreatic cancer **(B)**. Left: cell type distributions. Middle: cells colored by tumor grade. Right: cells colored by ITH score. Sample-level metrics are mapped to all cells from each sample.

**Fig. S2. Associations with clinical features and therapy response in single-cell datasets. (A-F)** UMAP visualizations in ovarian cancer **(A),** Head and neck cancer **(B),** Pancreatic cancer **(C),** two lung cancer cohorts **(D-F)**. Left: cells colored by clinical features. Right: cells colored by ITH score. Sample-level metrics are mapped to all cells from each sample.

**Fig. S3. ITH scores correlate with tumor progression and aggressiveness. (A)** In colorectal, kidney, gastric, and pan-cancer datasets, ITH scores were higher in tumor specimens than in normal tissues. **(B)** In kidney cancer, the more aggressive clear cell renal cell carcinoma (KIRC) showed higher ITH scores than the more indolent papillary renal cell carcinoma (KIRP).

**Fig. S4. ITH scores correlate with tumor progression and aggressiveness. (A)** ITH scores across six clustering resolutions in SKCM. Each line represents a sample; ITH scores increased with higher resolutions. Boxplots indicate median and IQR; jittered points represent individual sample value. **(B)** ROGUE-based heterogeneity scores across the same resolutions. ROGUE decreased with increasing resolution and showed lower consistency across samples than QeITH. Boxplots indicate median and IQR; jittered points represent individual sample value. **(C)** Correlations between QeITH and ROGUE scores at each resolution. Correlation strength varied across resolutions. Each panel represents a different clustering resolution; points represent individual samples; lines indicate linear trends. The Spearman correlation coefficients (*ρ*) and *P*-values are shown.

**Fig. S5. Kaplan-Meier survival analysis according to ITH scores. (A)** Kaplan-Meier survival curves comparing patients with low ITH scores (bottom tertile) and high ITH scores (top tertile) across multiple cancer types. Patients with low ITH scores exhibited significantly better survival outcomes across all four endpoints: OS, DSS, PFI, and DFS. Log-rank test *P*-values are shown. **(B)** Forest plot showing multivariable Cox regression results for OS across cancer types. Hazard ratios (HR) with 95% CI are shown. **(C)** Forest plot showing stage-stratified Cox models for OS, confirming robustness (HR = 1.48, 95% CI: 1.39–1.57). **(D)** Kaplan-Meier curves showing OS for cancer type groups stratified by median ITH and MAD. **(E)** Heatmap showing QeITH with five algorithms (MATH, PhyloWGS, ABSOLUTE, DEPTH2, DITHER) across clinical associations. Cancer types with FDR < 0.05 are indicated by asterisks. Point size and color represents -log10(*P*-value). **(F)** Dot plots showing Spearman correlations between QeITH and genomic ITH metrics (MATH, PhyloWGS) across individual cancer types. Each dot represents the correlation estimate (*ρ*) for a specific cancer type; dot size reflects the magnitude of the correlation. Cancer types with FDR < 0.05 are indicated by asterisks. **(G)** Robustness of QeITH to methodological choices. Top: Pairwise correlation matrix showing agreement between QeITH scores calculated using different methodological approaches: zero-thresholding (original), global shift method for negative value handling, rank-based quantile normalization, and square normalization. Upper triangle displays Spearman correlation coefficients; lower triangle shows scatter plots with fitted lines. All methods showed high correlations with the original approach. Bottom: Bar plot showing mean correlation between ITH scores calculated using random subsets of gene sets (ranging from 6 to 13 gene sets) and the original 14-gene-set score. Error bars indicate 95% confidence intervals; red dashed line indicates *ρ* = 0.90. The Spearman correlation coefficients (*ρ*) and *P*-values are shown.

**Fig. S6. Correlation of ITH scores with tumor neoantigen burden and *PD-L1* expression. (A)** Boxplots showing ITH score distributions in responders versus non-responders across two SKCM cohorts and one NSCLC cohort. Boxplots indicate median and IQR; points represent individual sample value. Higher ITH scores were significantly associated with improved response to immune checkpoint blockade in all three cohorts. Two-tailed Mann-Whitney *U* test *P*-values are shown. **(B)** ITH scores showed significant positive correlations with tumor neoantigen burden in 11 cancer types. **(C)** ITH scores showed significant positive correlations with *PD-L1* expression levels in 31 cancer types. The Spearman correlation coefficients (*ρ*) and *P*-values are shown.

**Fig. S7. Spatial heterogeneity of ITH scores in tumor invasion and metastasis. (A)** In breast cancer (BRCA): Cell type composition determined by CARD deconvolution (**left**) and spatial mapping of ITH scores (**center**) across tissue regions annotated based on the original study (**right**). Half-eye plots show ITH score distributions across regions. ITH scores were significantly higher in lymphocyte-infiltrated invasive tumor regions compared to ductal carcinoma in situ (DCIS). **(B)** In pancreatic cancer: Cell type composition across primary tumor, liver metastasis, and peritoneal metastasis from the same patient (**top**). Corresponding ITH scores are shown in a heatmap (**bottom**). The raincloud plot indicates significantly lower ITH scores in the primary tumor compared to metastatic sites. Half-violins show density distributions; boxplots indicate median and IQR; jittered points represent individual spot values.
