# Supplementary figures and images for: QeITH: Quantifies Tumor Ecosystem Heterogeneity to Predict Cancer Progression and Treatment Benefit
Source: Comput Struct Biotechnol J. 2026 Jun 18;35(1):0061. doi: 10.34133/csbj.0061 (PMC13276245; doi:10.34133/csbj.0061)

Fig. S1

A

GSE176078 - Breast cancer

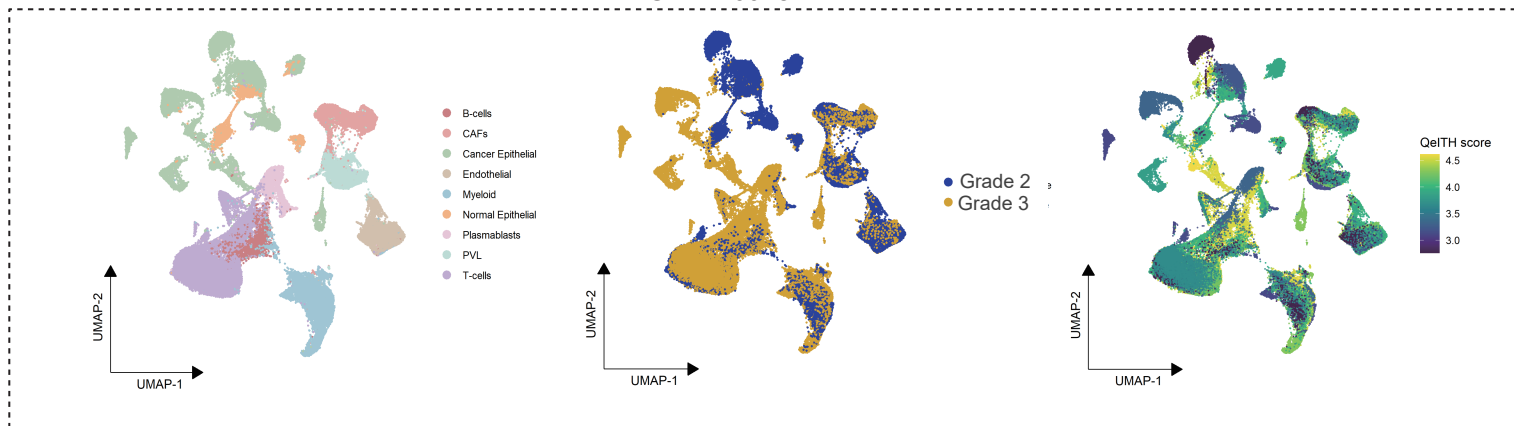

B

GSE263733 - Pancreatic cancer

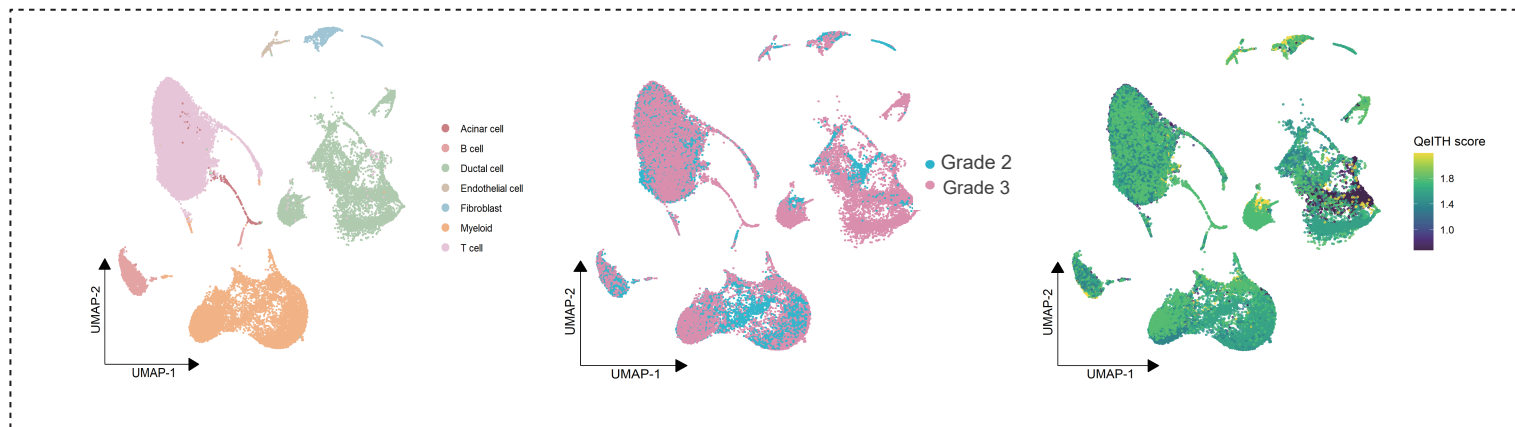

Supplement: Supplementary 1 — Figs. S1 to S7 Tables S1 to S5 [file csbj.0061.f1.zip › FIG.S1.pdf]

Fig. S5

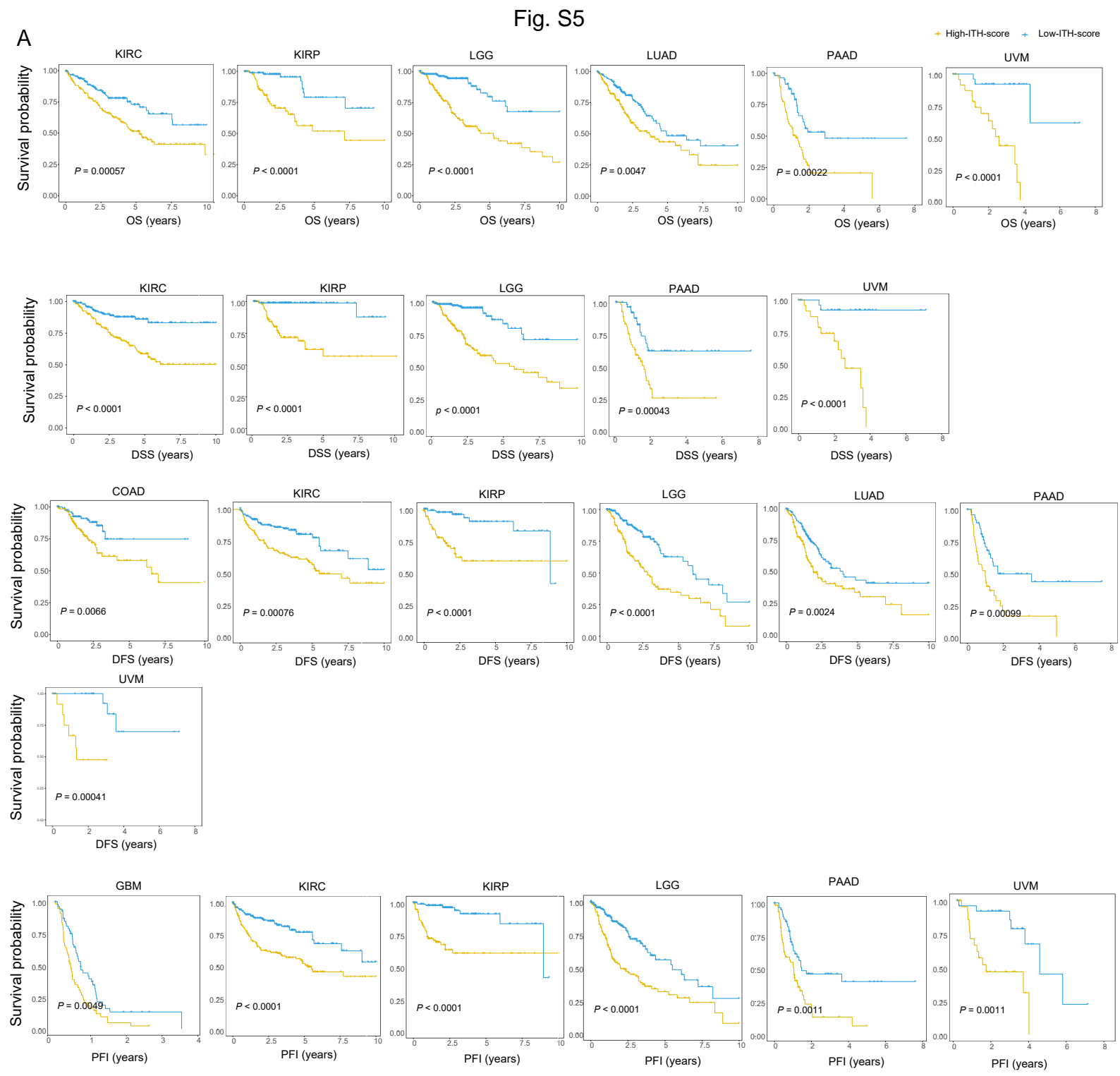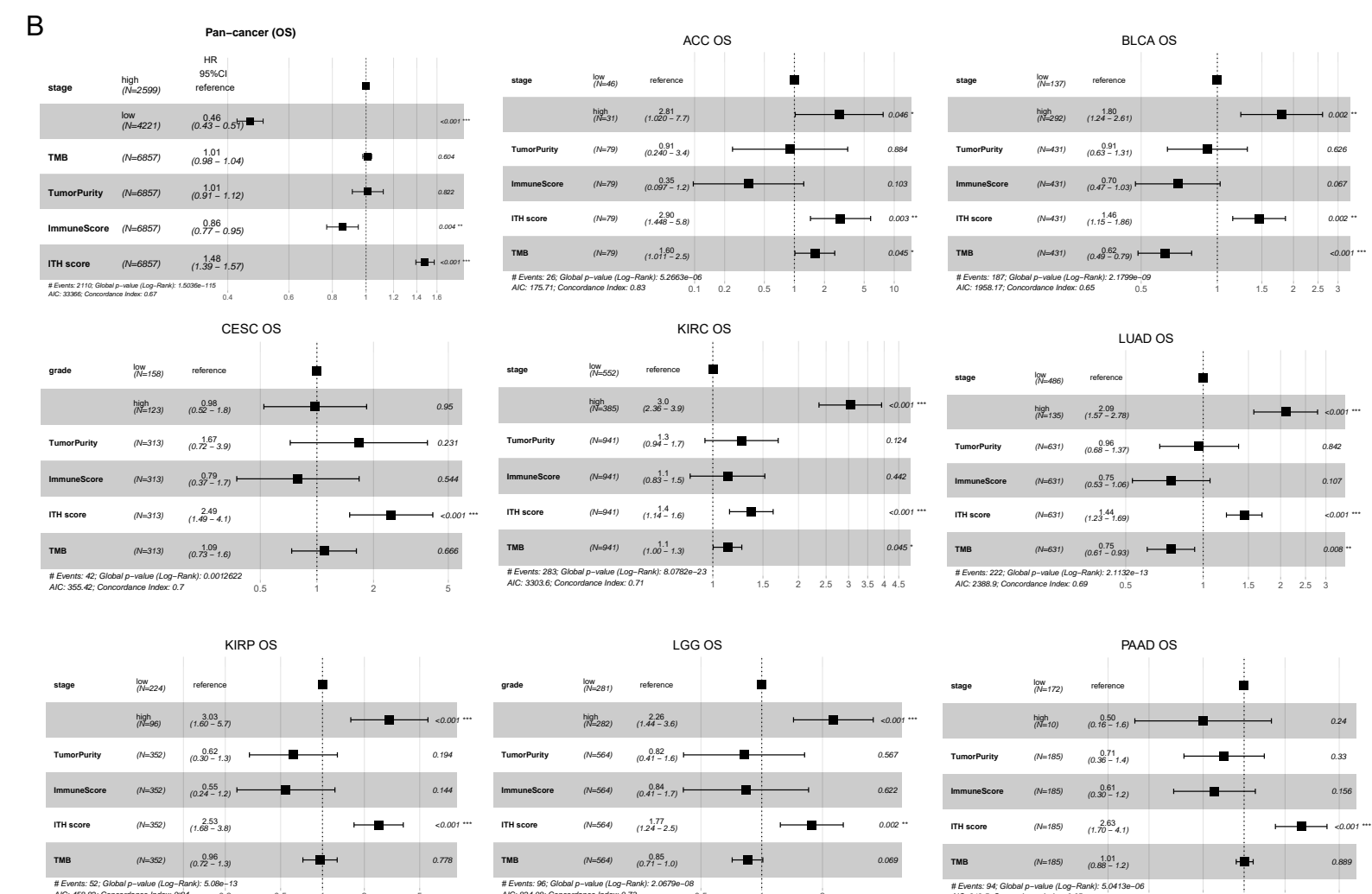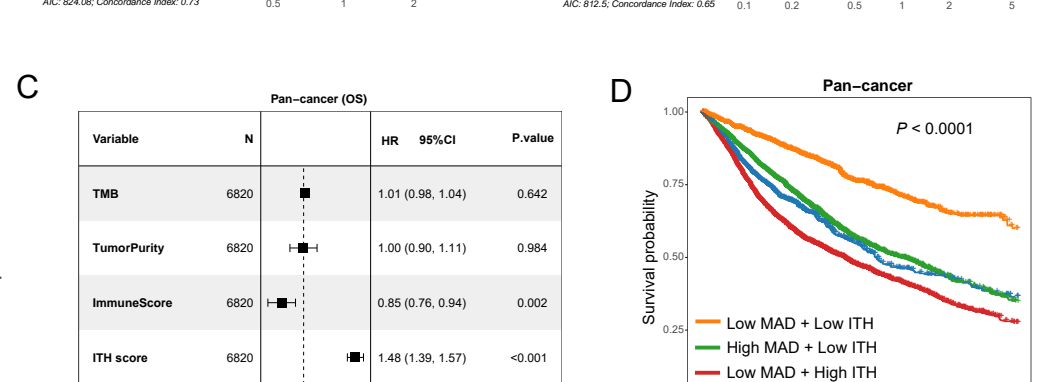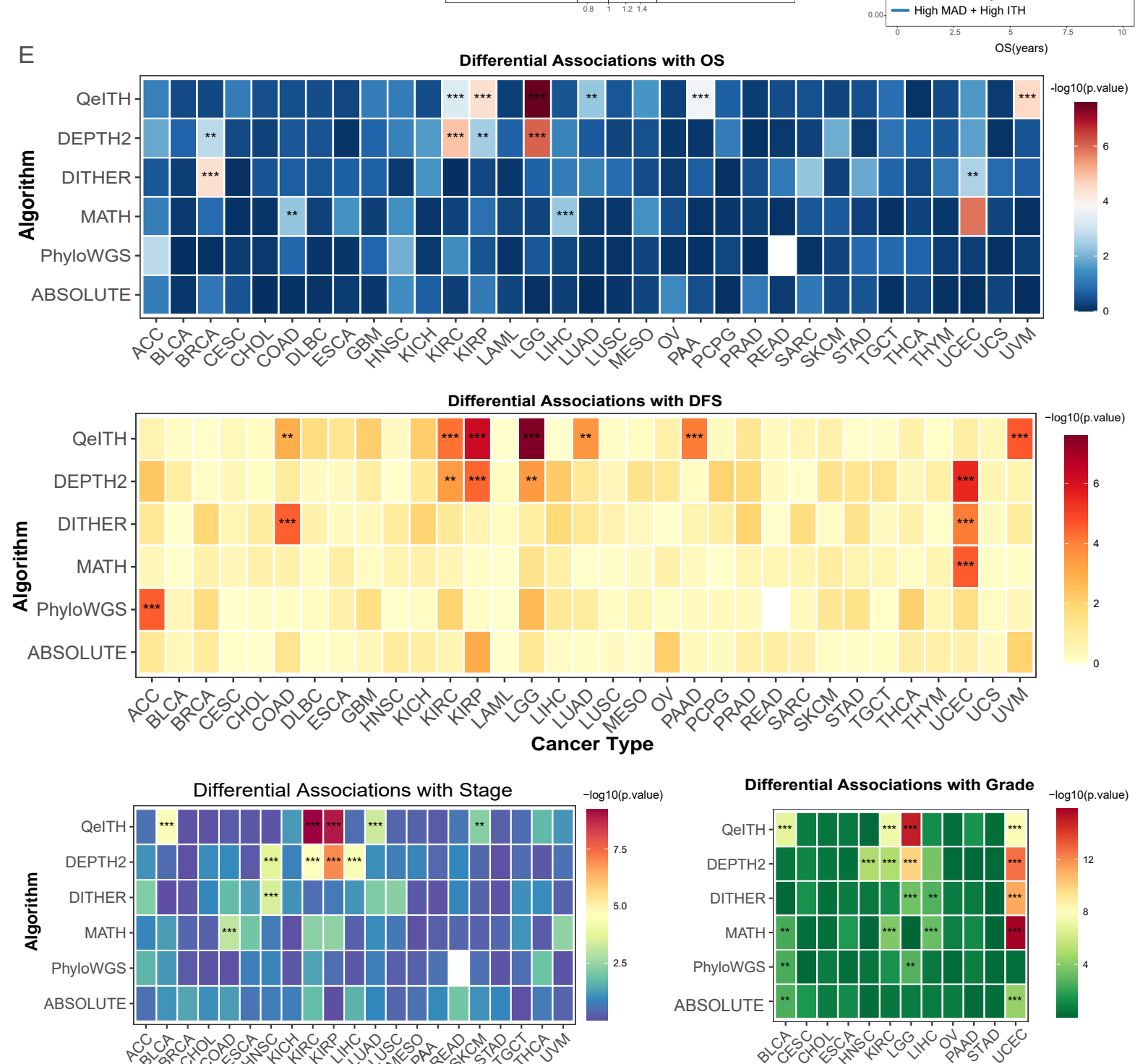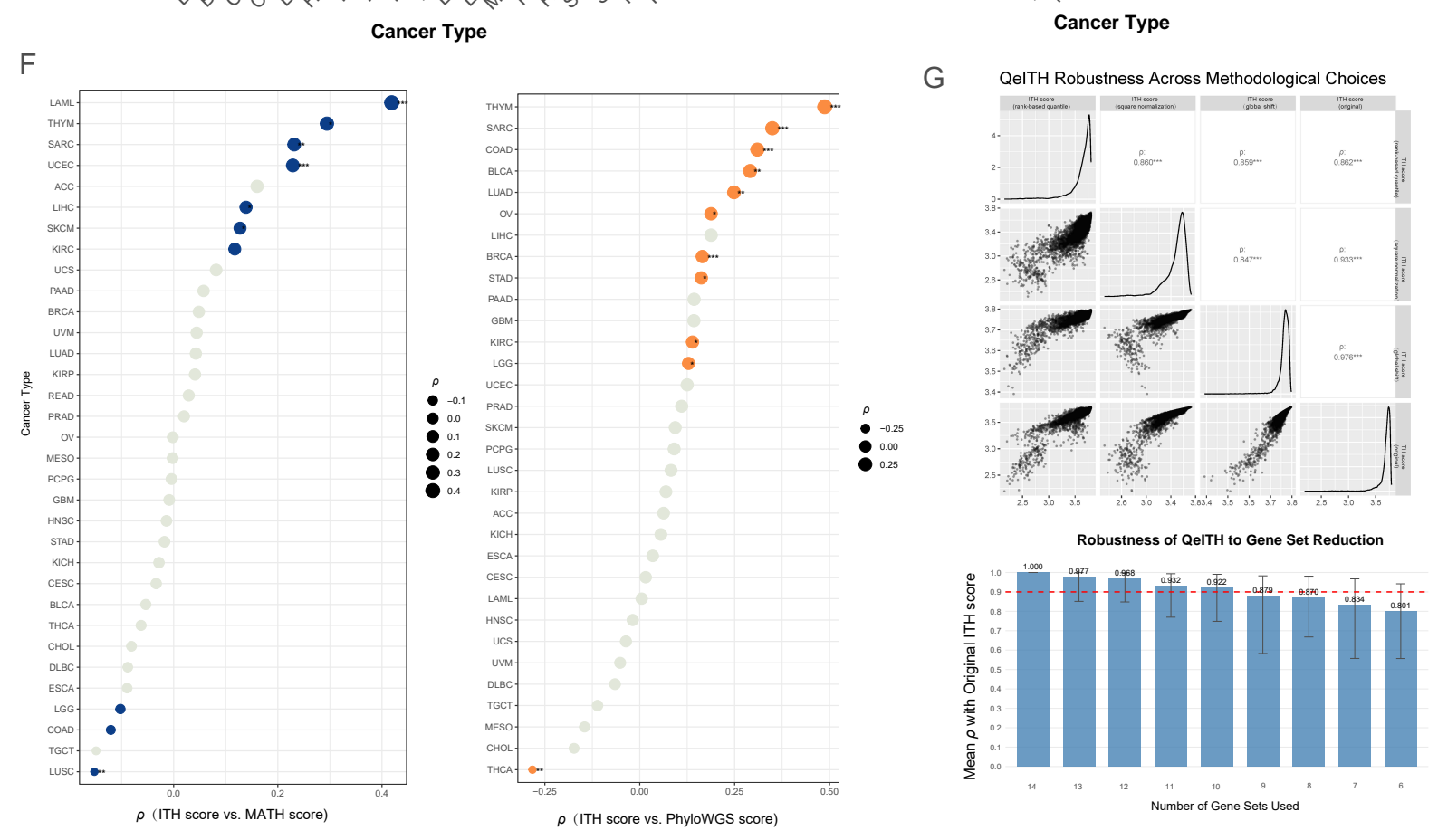

Supplement: Supplementary 1 — Figs. S1 to S7 Tables S1 to S5 [file csbj.0061.f1.zip › FIG.S5.pdf]

Fig. S6

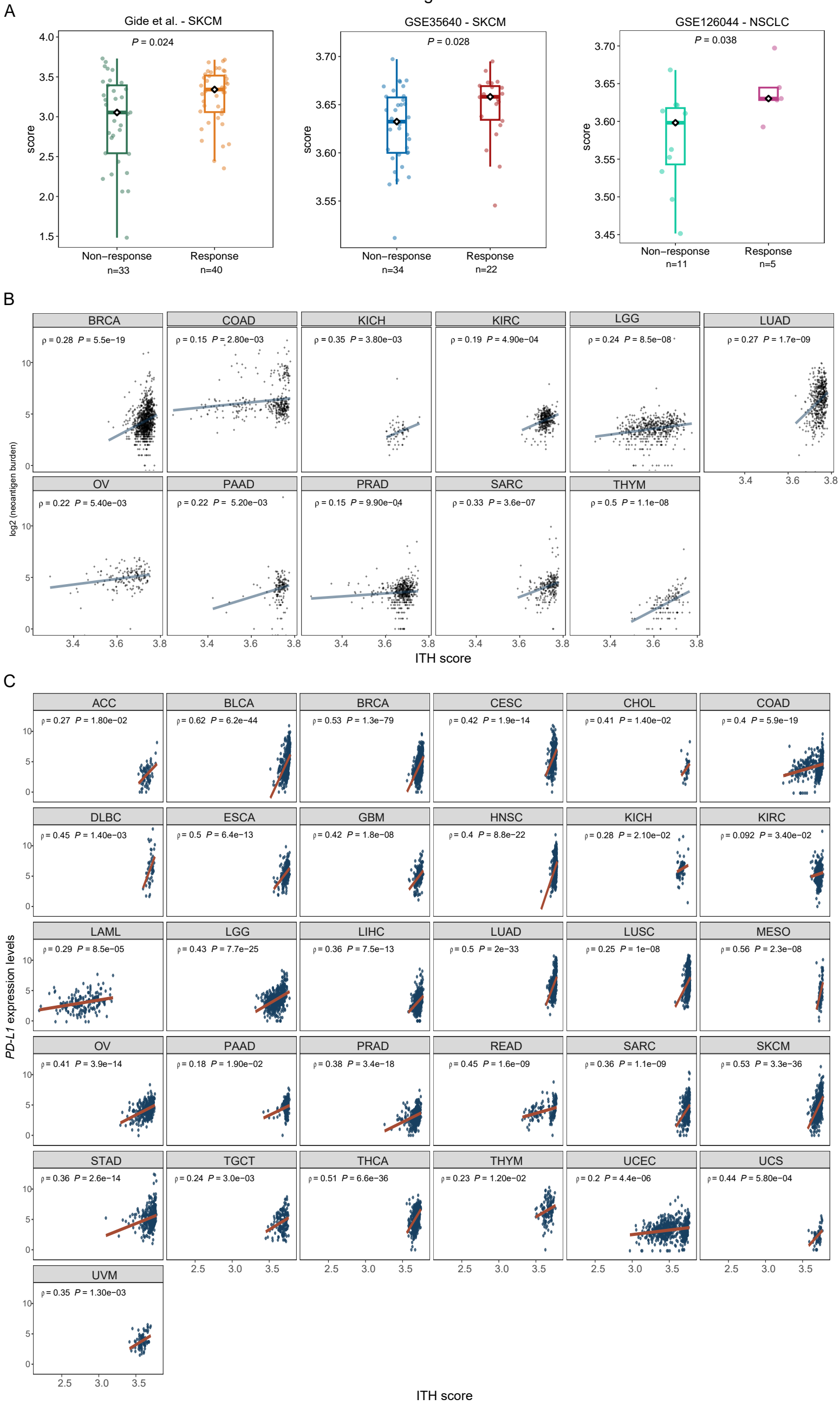

Supplement: Supplementary 1 — Figs. S1 to S7 Tables S1 to S5 [file csbj.0061.f1.zip › FIG.S6.pdf]

Fig. S7

A

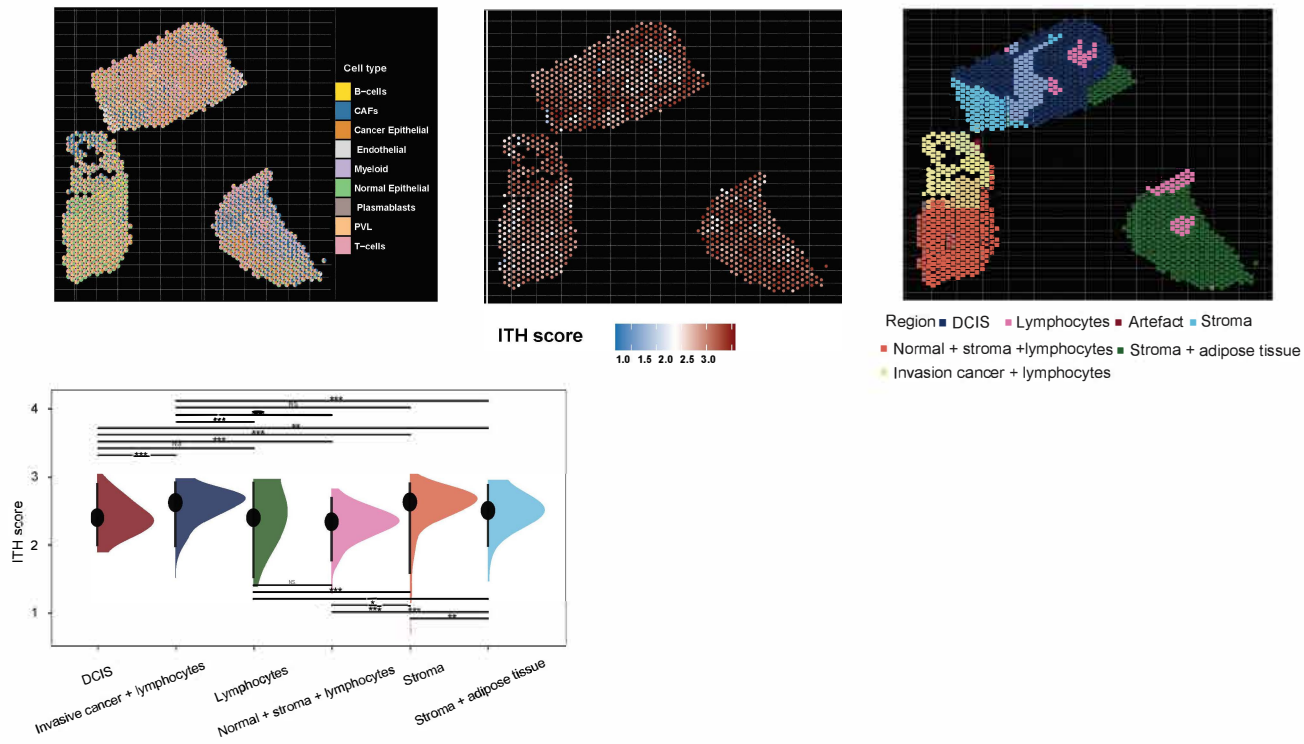

B

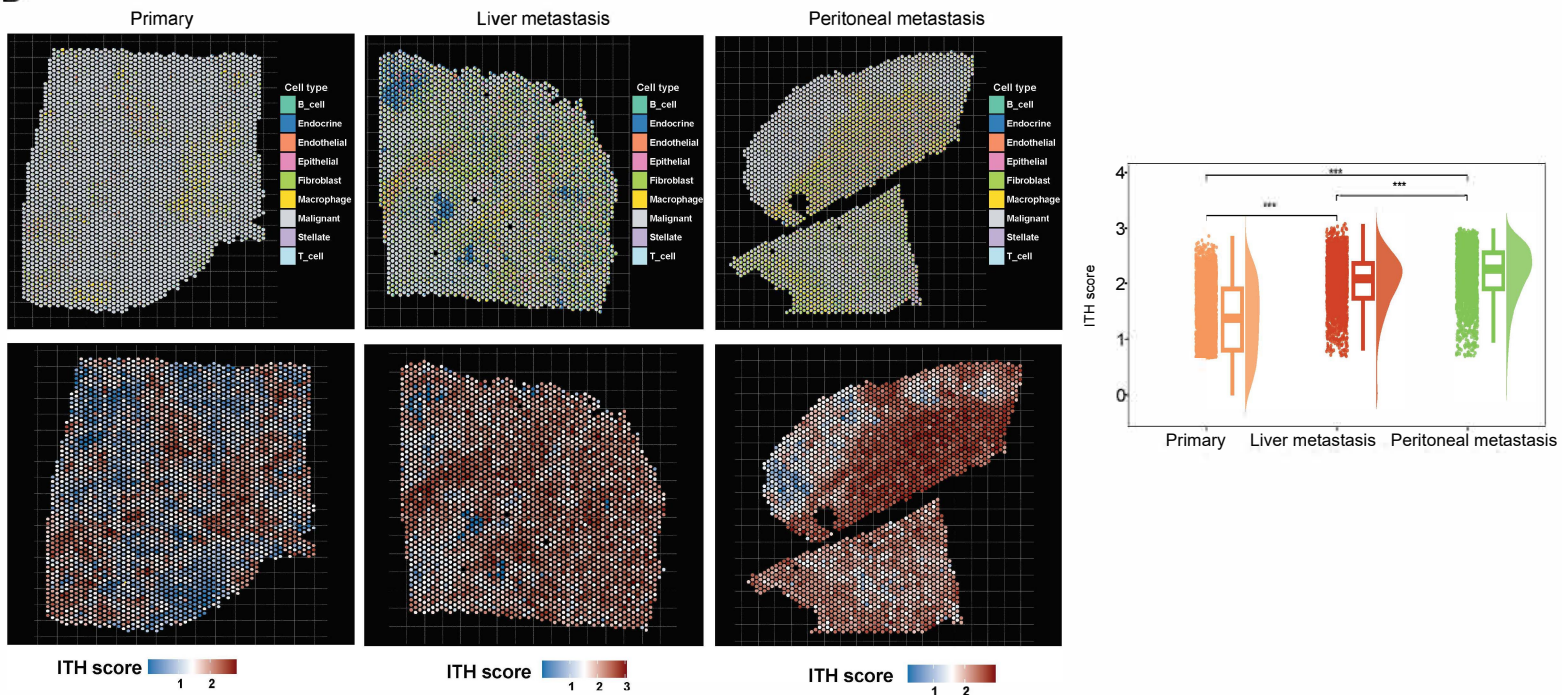

Supplement: Supplementary 1 — Figs. S1 to S7 Tables S1 to S5 [file csbj.0061.f1.zip › FIG.S7.pdf]
